# Chromatin and Single-Cell RNA-Seq Profiling Reveal Dynamic Signaling and Metabolic Transitions during Human Spermatogonial Stem Cell Development

**DOI:** 10.1016/j.stem.2017.09.003

**Published:** 2017-10-05

**Authors:** Jingtao Guo, Edward J. Grow, Chongil Yi, Hana Mlcochova, Geoffrey J. Maher, Cecilia Lindskog, Patrick J. Murphy, Candice L. Wike, Douglas T. Carrell, Anne Goriely, James M. Hotaling, Bradley R. Cairns

**Affiliations:** 1 Howard Hughes Medical Institute, Department of Oncological Sciences and Huntsman Cancer Institute, University of Utah School of Medicine, Salt Lake City, UT 84112, USA; 2 MRC Weatherall Institute of Molecular Medicine, Radcliffe Department of Medicine, University of Oxford, Oxford OX39DS, UK; 3 Department of Immunology, Genetics and Pathology, Science for Life Laboratory, Uppsala University, 751 85 Uppsala, Sweden; 4 Department of Surgery (Andrology/Urology), Center for Reconstructive Urology and Men’s Health, University of Utah Health Sciences Center, Salt Lake City, UT 84122, USA

**Keywords:** human spermatogonial stem cells, spermatogenesis, DNA methylation, open chromatin, pluripotency, single-cell RNA-seq, hormone receptors, metabolism

## Abstract

Human adult spermatogonial stem cells (hSSCs) must balance self-renewal and differentiation. To understand how this is achieved, we profiled DNA methylation and open chromatin (ATAC-seq) in SSEA4^+^ hSSCs, analyzed bulk and single-cell RNA transcriptomes (RNA-seq) in SSEA4^+^ hSSCs and differentiating c-KIT^+^ spermatogonia, and performed validation studies via immunofluorescence. First, DNA hypomethylation at embryonic developmental genes supports their epigenetic “poising” in hSSCs for future/embryonic expression, while core pluripotency genes (*OCT4* and *NANOG*) were transcriptionally and epigenetically repressed. Interestingly, open chromatin in hSSCs was strikingly enriched in binding sites for pioneer factors (*NFYA/B*, *DMRT1*, and hormone receptors). Remarkably, single-cell RNA-seq clustering analysis identified four cellular/developmental states during hSSC differentiation, involving major transitions in cell-cycle and transcriptional regulators, splicing and signaling factors, and glucose/mitochondria regulators. Overall, our results outline the dynamic chromatin/transcription landscape operating in hSSCs and identify crucial molecular pathways that accompany the transition from quiescence to proliferation and differentiation.

## Highlights

• Open chromatin in hSSCs correlates with pioneer factors and hormone receptors
• hSSC differentiation involves four sequential cellular/developmental states
• Key transitions involve the cell cycle, transcription factors, signaling, and metabolism

## Graphical Abstract

**Figure:**
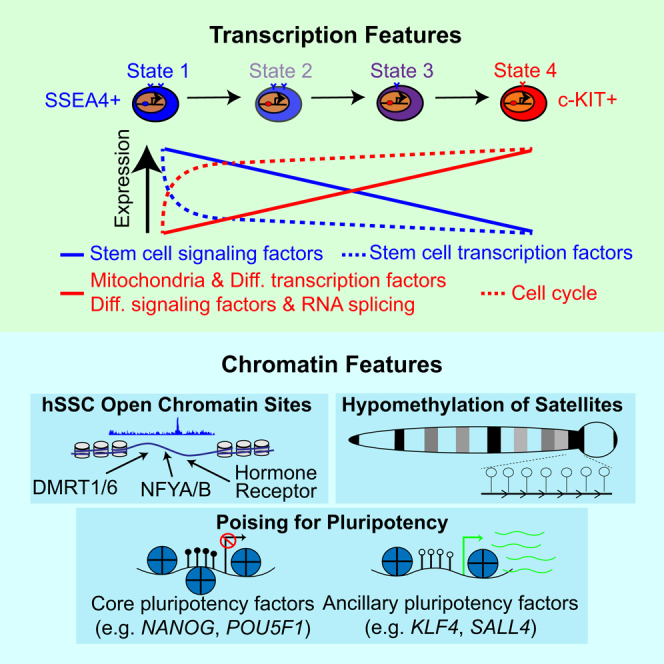

## Teaser

Cairns and colleagues show that human spermatogonial stem cells (hSSCs) bear unique DNA methylation and open chromatin landscapes, which may enable proper development, niche responsiveness, and “poised” pluripotency. Interestingly, single-cell transcriptome and immunofluorescence analyses reveal four cellular states, spanning from quiescent hSSCs to proliferating, metabolically active, differentiating spermatogonia.

## Introduction

Human adult spermatogonial stem cells (hSSCs) are the germline stem cells of adult males and display a set of fascinating stem cell properties (Guo et al., 2014b, Kanatsu-Shinohara and Shinohara, 2013, Payne, 2013). First, they must maintain a germline identity and a paternal-specific pattern of epigenetic imprinting. Second, through communication with their testicular niche, they delicately balance self-renewal with differentiation long-term, to avoid exhaustion and allow lifelong gametogenesis. Third, although they are stem cells, hSSCs are “unipotent,” despite stages of amplification and differentiation, their developmental trajectory culminates in the formation of only one cell type—mature sperm.

The mechanism underlying unipotency in mouse SSCs/germline may involve the inhibition of pluripotency, as key pluripotency and developmental genes are packaged into a “poised” chromatin that imposes silencing, while also enabling future activation (Erkek et al., 2013, Hammoud et al., 2014, Lesch et al., 2013). In support, SSCs from mice can efficiently convert to multipotent germline stem cells in culture (and re-express high *Oct4* and *Nanog*), suggesting a facile “unipotent-to-pluripotent” transition (Guan et al., 2006, Kanatsu-Shinohara et al., 2004). However, whether these molecular features are conserved in hSSCs and differentiating spermatogonia is unknown, and of high interest.

Testicular niche cells include Sertoli cells and Leydig cells (Kanatsu-Shinohara et al., 2012, Yoshida et al., 2007), which provide important growth factors, hormones, and chemokines—which either reinforce the SSC state or enable transition to cells committing to differentiation (termed spermatogonia) and coordinate the additional stages of spermatogenesis. Extensive studies of mouse SSCs and niche cells have provided a wealth of information on the key signaling systems, transcriptional drivers, and diagnostic markers of germline stem cell states (Brinster and Zimmermann, 1994, Kanatsu-Shinohara and Shinohara, 2013). For example, the transcription factors Id4 and Sall4, and the signaling factors Gfra1 and certain Fgf-family receptors are strongly correlated with SSC status in the mouse. In counter distinction, the cell-surface marker Kit and the transcription factors Sohlh1/2 are associated with differentiating spermatogonia (Chan et al., 2014, Hammoud et al., 2014, Kanatsu-Shinohara and Shinohara, 2013). Although more limited, related studies in humans have revealed both similarities and differences with mice, prompting more detailed comparative studies (Boitani et al., 2016). Regardless, studies across mammals suggest a complex differentiation pathway that likely involves heterogeneity at both the SSC and differentiation stages (Hammoud et al., 2015, Hara et al., 2014, Hermann et al., 2015, Kanatsu-Shinohara and Shinohara, 2013, Klein et al., 2010, von Kopylow et al., 2016). This heterogeneity is not revealed in standard genomics approaches that typically involve analysis of bulk material and cell isolation procedures using a single surface marker but is well addressed through single-cell approaches and analytical methods.

Here, we aim to define the DNA methylation (DNAme), chromatin, and transcription states of adult hSSCs, in order to understand how transcription, signaling, and metabolic states transition during hSSC differentiation. Notably, we find open chromatin enriched at the binding sites for hormone receptors and potential pioneer factors, which may prime hSSCs for hormonal response. Most importantly, our single-cell RNA sequencing (scRNA-seq) analysis reveals the existence of four distinct cellular states during the transition from hSSCs to differentiating c-KIT^+^ spermatogonia, delineating a potential developmental trajectory for hSSCs. Additionally, we used immunofluorescence to directly visualize the protein expression of a subset of the differentially expressed genes identified by scRNA-seq, allowing us to validate key genomics findings in situ. Taken together, we define the dynamic hSSC chromatin/transcriptional landscape in hSSCs and delineate key transcriptional, metabolic, and signaling pathways underlying the transition of hSSCs from quiescence to proliferation and differentiation.

## Results

### Genomic Profiling of SSEA4^+^ hSSCs and c-KIT^+^ Spermatogonia

**Figure 1:**
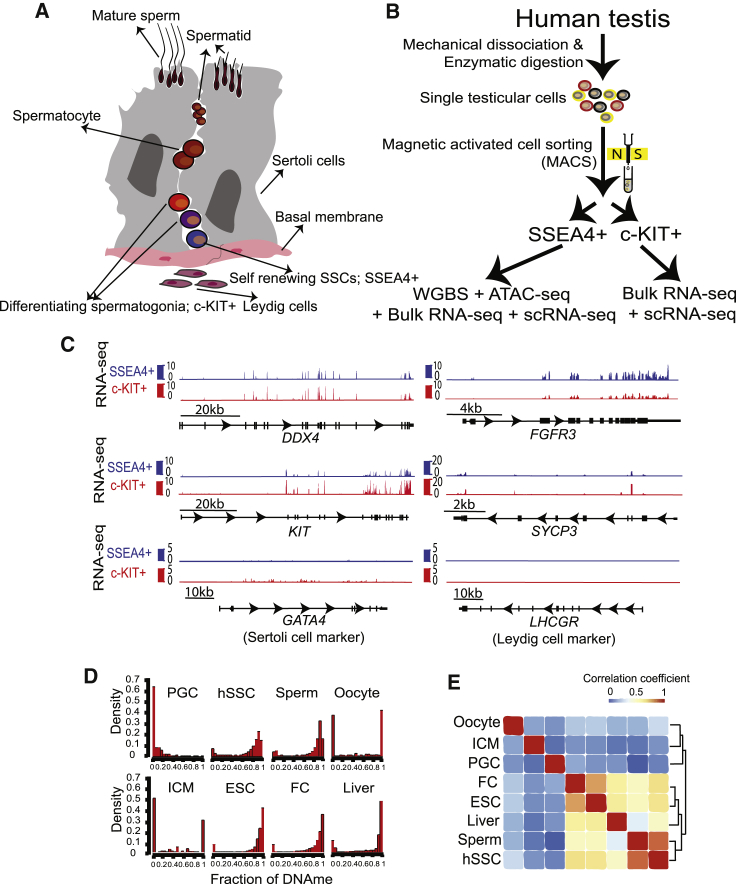
Genomic Profiling of Human Spermatogonial Stem Cells (A) Schematic illustration of human adult male germline development and niche, depicting a small section of the seminiferous tubule. (B) Experimental workflow in this study. sc, single cell; WGBS, whole-genome bisulfite sequencing. (C) Expression profiles of selected key genes following the enrichment procedure with SSEA4 (blue) or c-KIT (red). Browser snapshots of *DDX4* (germ cell marker), *FGFR3* (hSSC marker), *KIT* and *SYCP3* (differentiating spermatogonia marker), *GATA4* (Sertoli cell marker), and *LHCGR* (Leydig cell marker). The intron/exon (box) genomic structure of each gene is shown in black. (D) Distribution of DNAme in human PGCs, hSSCs, sperm, egg, ICMs (inner cell mass), ESCs, FC (frontal cortex), and liver. Human PGC and liver methylation data are from Guo et al. (2015); ICM and FC methylation data are from Guo et al. (2014a); egg methylation data are from Okae et al. (2014); ESC methylation data are from Gifford et al. (2013). (E) Hierarchical clustering of correlation of global DNAme in human PGCs, hSSCs, sperm, egg, ICMs, ESCs, FC, and liver. See also Figures S1 and S2.

Multiple lines of evidence support SSEA4 as a marker of hSSCs, and c-KIT as a marker of spermatogonia committing/committed to differentiation (Izadyar et al., 2011, Valli et al., 2014a) (Figure 1A). Both SSEA4^+^ hSSCs and subsequent c-KIT^+^ spermatogonia reside within an intermediate compartment formed between the basal lamina and cell junctions formed by Sertoli cells, which allow migrating spermatocytes to pass to the adluminal compartment (Figure 1A). We isolated SSEA4^+^ hSSCs and c-KIT^+^ spermatogonia from whole adult human testis, from five patients experiencing idiopathic pain, not involving cancer or major inflammation. Following a set of rinsing, dissection, digestion, and filtering steps, we used magnetic activated cell sorting (MACS) to acquire highly enriched populations, which were used for either bulk approaches or additional single-cell isolation. Bulk approaches included profiling DNAme (via whole-genome bisulfite sequencing [WGBS]), chromatin accessibility (via ATAC sequencing [ATAC-seq]), and transcriptome (via RNA-seq). To refine our understanding of how hSSCs differentiate into spermatogonia, we also performed scRNA-seq in isolated SSEA4^+^ and c-KIT^+^ cells (Figure 1B).

We first evaluated the the purity and identity of the sorted cell fractions by flow cytometry (Figures S1A and S1B) and immunofluorescence (Figure S1C), which revealed that SSEA4 enrichment generates cell populations that are >90% SSEA4^+^. Furthermore, certain genomics results (previewed here) also strongly support the efficiency of our cell enrichment procedures. First, our DNAme profiling of SSEA4^+^ hSSCs revealed clear DNA hypomethylation of meiosis-related genes and paternal imprinted sites, and high methylation at maternal imprinted sites (Figures S1E and S2). Second, our transcriptome data showed the expected expression patterns of key markers from mouse and human studies: for example, the germ cell marker (*DDX4*) was expressed in both SSEA4^+^ and c-KIT^+^ cells, the self-renewal marker (*FGFR3*) was upregulated in SSEA4^+^ hSSCs, differentiating markers (*KIT* and *SYCP3*) were upregulated in c-KIT^+^ spermatogonia, and known markers of Sertoli cells (*GATA4*) and Leydig cells (*LHCGR*) were extremely low or absent (Figure 1C). Taken together, our genomic data (with additional examples below) confirmed high enrichment and sorting efficiency of germline stem cells.

### DNAme Profiling of SSEA4^+^ hSSCs

We began by examining DNAme of bulk hSSCs, as DNAme patterning and reprogramming can help guide (or restrict) gene expression and stem cell development (Smith and Meissner, 2013). Notably, our DNAme datasets (bisulfite conversion efficiency >99%) revealed that hSSC DNAme profiles were nearly identical to those of mature sperm (r = 0.95) (Figures 1D and 1E) at both promoter and putative enhancer sites (Figure S1D) as well as imprinted loci (Figure S2), demonstrating that DNAme does not markedly change between adult hSSCs and mature sperm, consistent with results in the mouse (Hammoud et al., 2014). Therefore, DNAme in c-KIT^+^ spermatogonia was not examined.

### ATAC-Seq Reveals Open Chromatin at Binding Sites for Potential Pioneer Factors and Hormone Receptors

**Figure 2:**
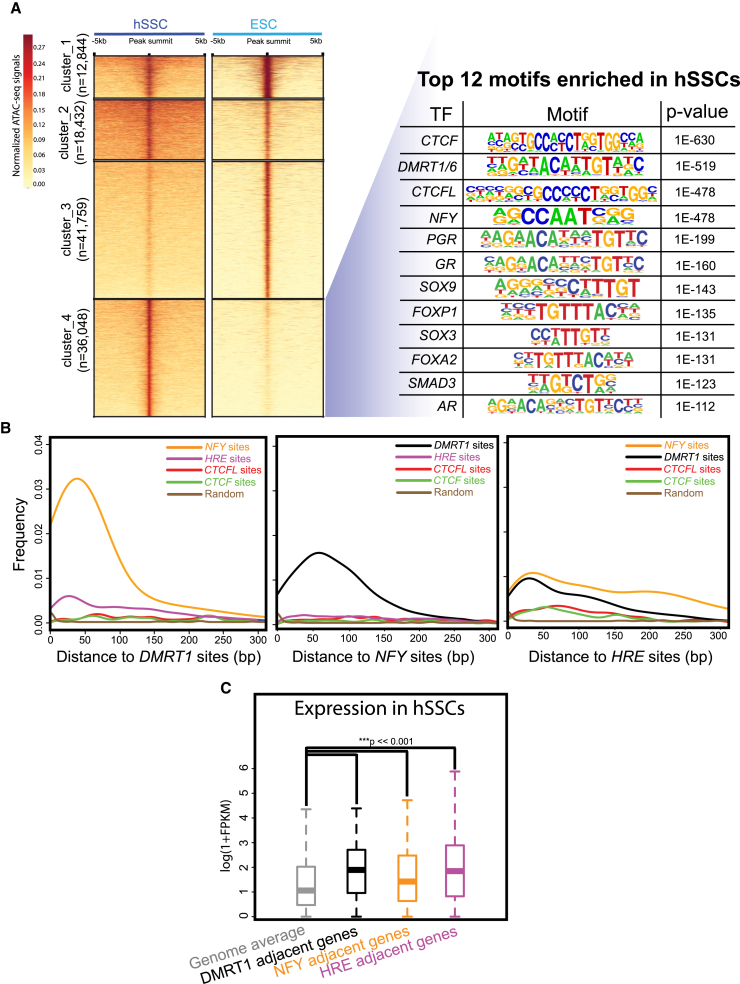
Unique Chromatin Landscape in hSSCs Revealed by ATAC-Seq (A) Heatmap of k-means clustering (n = 4) showing ATAC-seq signals at ESC and hSSC peaks and motifs enriched in each cluster. (B) Distance between NFY sites, DMRT1 sites, and HRE sites. (C) Expression of genes adjacent (within 10 kb) to DMRT1 sites, NFY sites, and HRE sites are specifically upregulated in hSSCs. See also Figures S3 and S4.

Here, to delineate the chromatin landscape and identify potential drivers of the hSSC transcriptome, we analyzed hSSCs by ATAC-seq (two replicates from two patients, r > 0.8; Figure S3A) and compared to embryonic stem cells (ESCs). After peak calling to define open regions, we performed clustering analysis, which revealed peaks shared between hSSCs and ESCs (clusters 1 and 2), ESC-specific peaks (cluster 3), and hSSC-specific peaks (cluster 4) (Figure 2A). Properties of shared peaks include enrichment around promoter sites (Figures S3B and S3C). Next, we applied motif discovery analyses to characterize binding motifs specifically enriched in open chromatin (ATAC-seq sites) of SSEA4^+^ hSSCs (Figure 2A). Interestingly, this analysis reveals binding sites for multiple factors in the unfiltered top 12 list that included *CTCFL/BORIS*, *DMRT1*, *NFYA*/*B* (pioneer factors implicated in early embryo chromatin landscape formation) (Lu et al., 2016), the hormone receptor element (HRE, recognized by *PGR* (progesterone receptor), *GR* (glucocorticoid receptor; *NR3C1*), and *AR* (androgen receptor)), as well as FOX factors and SOX-family factors (Figure 2A). Furthermore, we often found NFY and DMRT1 binding sites in very close proximity and observed a detectable bias for these sites to be near HRE elements (Figure 2B). Interestingly, we observed upregulation of genes located within 10 kb from DMRT1, NFYA/B or HRE binding sites (Figure 2C), with accompanying DNA hypomethylation tightly centered around DMRT1 and NFYA/B binding sites (Figure S3F). This finding raises the possibility that the hSSC chromatin and transcriptional landscapes are markedly influenced by hormone receptors and the pioneer factors NFYA/B and DMRT1, leading to upregulation of adjacent genes.

### Methylation and Chromatin Status of Repeat Elements in hSSCs

Regulation of repeat elements is a major feature of germline gene regulation (Tang et al., 2016). As expected, DNAme revealed that all major classes of repeat elements in hSSCs (e.g., LINE, SINE, and LTR) were highly methylated, at levels similar to those observed in somatic cells. However, unlike the situation in ESCs and somatic cells, satellite elements were hypomethylated in hSSCs and sperm (Figure S4A), especially ACRO1 satellites (Figure S4B). ACRO1 expression was low in male and female germ cells and somatic cells but increased significantly in the early embryo (Figure S4C). As transcription of satellites in mouse early embryos is linked to chromocenter formation and paternal genome reprogramming (Probst et al., 2010), their DNA hypomethylation in the human male germline may help poise them for expression, to facilitate proper paternal genome re-organization in the early human embryos.

Since primordial germ cells (PGCs) undergo global DNA demethylation and activation of transposable elements (Gkountela et al., 2015, Guo et al., 2015, Tang et al., 2015), we examined DNAme and chromatin opening (ATAC-seq) at transposable elements, and their correlation with transcription in hSSCs. First, LTR elements in aggregate show moderate chromatin opening in hSSCs but not ESCs (Figure S4D). However, parsing the data reveals chromatin opening within three specific LTR sub-families: LTR12C, LTR12D, and LTR12E, which were associated with strong ATAC-seq signals and DNA hypomethylation in hSSCs (Figures S4E–S4G). Notably, all three LTRs were upregulated in hSSCs and oocytes but downregulated during early embryonic development and in somatic cells (Figure 4H). Moreover, motif finding analysis revealed the NFYA/B binding motif highly enriched in the three LTRs (Figures S4I–S4K). Thus, our data suggest a role for LTR12C, LTR12D, and LTR12E in the human germline, possibly via their regulation by NFYA/B.

### Poised Pluripotency and Meiotic Potential in hSSCs

**Figure 3:**
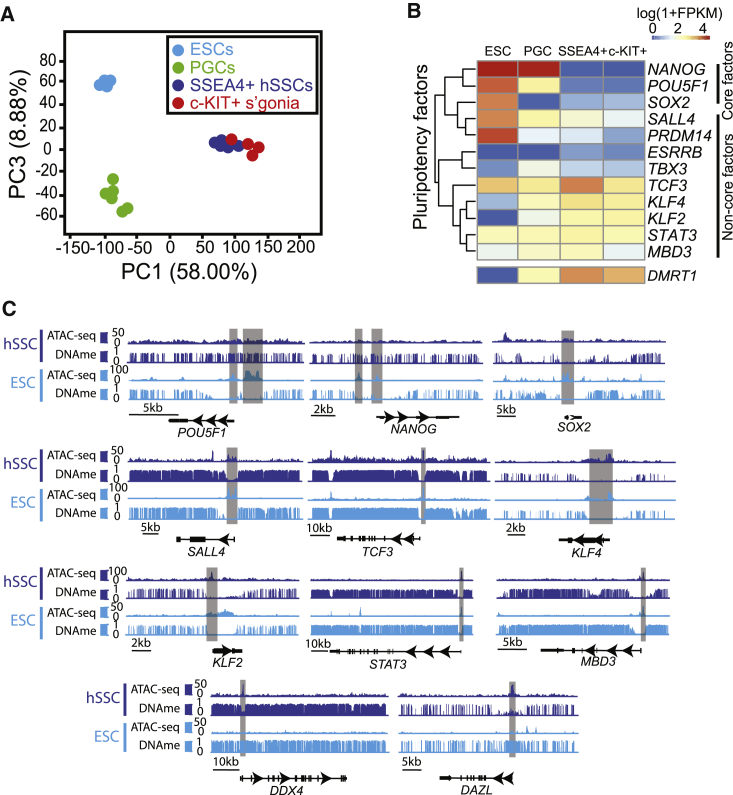
Chromatin Underlying Poised Pluripotency in hSSCs (A) Principal component analysis (PCA) of the transcriptome of SSEA4^+^ hSSCs, c-KIT^+^ spermatogonia, PGCs, and ESCs. Human PGC and ESC RNA-seq data are from Gkountela et al. (2015). (B) Hierarchical clustering of pluripotency-related factors from ESCs, PGCs, SSEA4^+^ hSSCs, and c-KIT^+^ spermatogonia. (C) Browser snapshots of ATAC-seq signals and DNAme at selected key genes. Note: *POU5F1*, *NANOG*, and *SOX2* encode core pluripotency factors; *SALL4*, *TCF3*, *KLF4*, *KLF2*, *STAT3*, and *MBD3* encode ancillary pluripotency factors; *DDX4* and *DAZL* are germ cell-specific markers. See also Figure S5.

To better define the unique molecular nature of hSSC states and determine the differences that may exist between germline and ESCs, we compared the RNA-seq profiling of bulk SSEA4^+^ hSSCs and c-KIT^+^ spermatogonia to each other, and to ESCs and PGCs (Gkountela et al., 2015). Principal component analysis (PCA) revealed that SSEA4^+^ hSSCs and c-KIT^+^ spermatogonia clustered near one another but were distant from both PGCs and ESCs (Figure 3A). For example, meiosis- and pluripotency-related genes clearly distinguish hSSCs and c-KIT^+^ cells from ESCs and PGCs (Figures S5C and S5D). While meiosis-related genes were repressed in early and late PGCs, they were gradually upregulated in hSSCs and spermatogonia. By contrast, the expression of core pluripotency genes (*OCT4*, *NANOG*, *SOX2*) was found to be low or undetectable in hSSCs and spermatogonia, although other pluripotency-related factors were expressed (Figure 3B) (Hackett and Surani, 2014). These distinct properties may underlie hSSCs and c-KIT^+^ cells unipotent potential. Furthermore, we found *DMRT1*, a key differentiation factor known to antagonize pluripotency in the mouse, expressed at markedly higher levels in hSSCs/spermatogonia than ESCs (Takashima et al., 2013) (Figure 3B).

To better understand the chromatin-transcription relationships for key spermatogenesis genes, we examined their chromatin and DNAme status in details (Figure 3C). Notably, we found that *POU5F1*/*OCT4* and *NANOG* promoters were fully methylated and showed no ATAC-seq signals, which likely explain their repressed status; however, the *SOX2* promoter was hypomethylated and exhibited ATAC-seq peaks, suggesting that other inhibitory mechanisms are involved in *SOX2* regulation. Furthermore, we found DNA hypomethylation at the promoters of the other pluripotency factors *KLF4*, *SALL4*, *TCF3*, *MBD3*, *STAT3*, and *KLF2*, along with ATAC-seq peaks, consistent with their activation in hSSCs. As a control, the promoters of germline-expressed genes (e.g., *DDX4* and *DAZL*) displayed open chromatin and DNA hypomethylation in hSSCs, while closed chromatin with full methylation was observed in ESCs, a finding consistent with the germ cell epigenetic/transcription status of hSSCs.

### Single-Cell Transcriptome Profiling

A subset of spermatogonia are both SSEA4^+^ and c-KIT^+^ (Izadyar et al., 2011), and we noted during the RNA-seq analysis of the bulk SSEA4-enriched cell fraction that *KIT* expression was detected at low levels (Figure 1C), raising the possibility that doubly positive cells might have been isolated during our enrichment/sorting procedure. We reasoned that these cells may represent transitioning cellular states of high interest, and that they could be properly profiled in single-cell formats. From a total of 175 single-cell datasets, 92 single cells (60 SSEA4^+^ and 32 c-KIT^+^) passed stringent filtering criteria. Consistent with our bulk RNA-seq results, all 92 expressed germline-specific genes and lacked somatic cell markers (Table S1). As expected, we observed markers associated with self-renewing hSSCs or differentiating spermatogonia preferentially expressed in SSEA4^+^ or c-KIT^+^ cells, respectively (Figure S5E).

**Figure 4:**
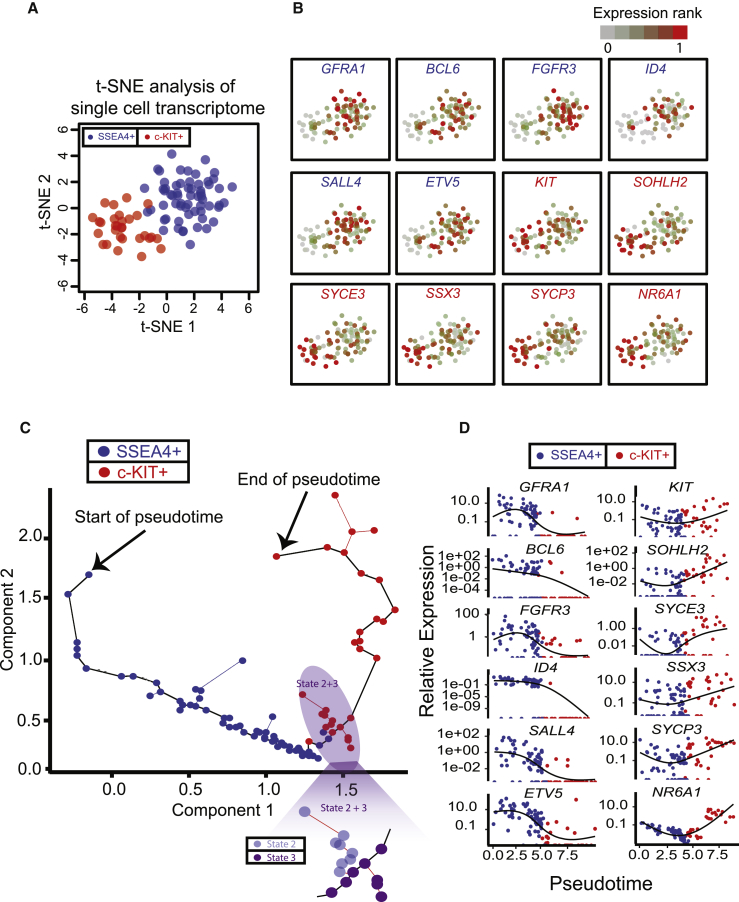
Single-Cell Transcriptome Analysis by t-SNE and Monocle (A) t-SNE analysis plot of single-cell transcriptome. t-SNE, t-distributed stochastic neighbor embedding. (B) Expression profiles of selected key genes in SSEA4-enriched or c-KIT-enriched single cells projected on the t-SNE plot. (C) Monocle analysis plot of scRNA-seq data, in which gene expression in multi-dimensional space is compressed to two dimensions/components. Most cells were positioned along a central branch, with two small branches emanating at the transition between SSEA4^+^ and c-KIT^+^ cells. The states assignment involved subsequent hierarchical clustering shown in Figure 6B. (D) Expression of selected key genes along pseudotime development. SSEA4^+^ (blue) or c-KIT^+^ (red) cells are projected along pseudotime. Genes associated with self-renewal are depicted on the left column, and genes associated with c-KIT^+^ differentiating cells along the right column. Note: depicted as compressed (log10) transformed expression data, and only 30%–70% of single cells typically provide non-zero expression of individual genes. See also Figure S5.

We first analyzed the data with t-distributed stochastic neighbor embedding (t-SNE). t-SNE analysis on filtered and normalized single-cell transcriptome data efficiently separated SSEA4^+^ hSSCs and c-KIT^+^ spermatogonia, with hSSCs on the top right and spermatogonia on bottom left (Figure 4A). We then projected gene expression patterns onto the t-SNE distribution. Here, we chose *GFRA1*, *BCL6*, *FGFR3*, *ID4*, *SALL4*, and *ETV5* as hSSC markers, and *KIT*, *SOHLH2*, *SYCE3*, *SSX3*, *SYCP3*, and *NR6A1* as spermatogonia markers, based on work in the mouse and results from our bulk datasets. We noticed a clear trend of higher hSSC marker expression in SSEA4-enriched cells and a clear trend of higher differentiating marker expression in c-KIT^+^ cells (Figure 4B). However, t-SNE also revealed exceptions to these trends, suggesting cellular heterogeneity—prompting additional approaches to exploit these discrepancies in order to identify developmental transitions.

To further distinguish SSEA4^+^ and c-KIT^+^ populations, and to discover potential intermediate/transitional states, we used Monocle (Trapnell et al., 2014). Monocle orders single cells with an unsupervised algorithm, without any prior knowledge of cell identity or isolation procedure/markers. First, Monocle compares all single-cell transcription datasets to each other in a multi-dimensional space, and compresses them into a two-dimension space for projection. A “minimal spanning tree” between samples is then created. Single cells within this two-dimension space (represented by dots) are ordered in “pseudotime,” with line connections between dots/cells showing a path of transcriptional relatedness that may also represent a developmental trajectory/timeline. Application of Monocle to our scRNA-seq datasets yielded a clear trajectory, revealing a large central branch, from which only a few minor branches emanated (Figure 4C). When the dots/cells were then colored by their surface selection markers, we observed a striking alignment of SSEA4-sorted cells to the large central branch at early pseudotime (Figure 4C, left), and alignment of the c-KIT^+^ cells to the large central branch at late pseudotime (Figure 4C, right), as well as to the central small branches emanating at the SSEA4/c-KIT transition. Thus, Monocle efficiently separated SSEA4-enriched cells from those selected with c-KIT and also singled out a subset of cells at the central branchpoint as candidate transitioning cells (addressed later).

We then examined how key markers were expressed along pseudotime, by providing plots of individual genes in every cell (Figure 4D). Notably, candidate hSSC markers (*GFRA1*, *BCL6*, *FGFR3*, *ID4*, *SALL4*, and *ETV5*) were highly expressed only at early pseudotime (Figure 4D, left), whereas candidate differentiating markers (*KIT*, *SOHLH2*, *SYCE3*, *SSX3*, *SYCP3*, and *NR6A1*) were more highly expressed at late pseudotime (Figure 4D, right). The consistent alignment of these human genes/markers in Monocle pseudotime to hSSC/spermatogonia development in the mouse strongly suggests that pseudotime reflects biological development.

### Signaling and Transcription Pathways along hSSC Development

**Figure 5:**
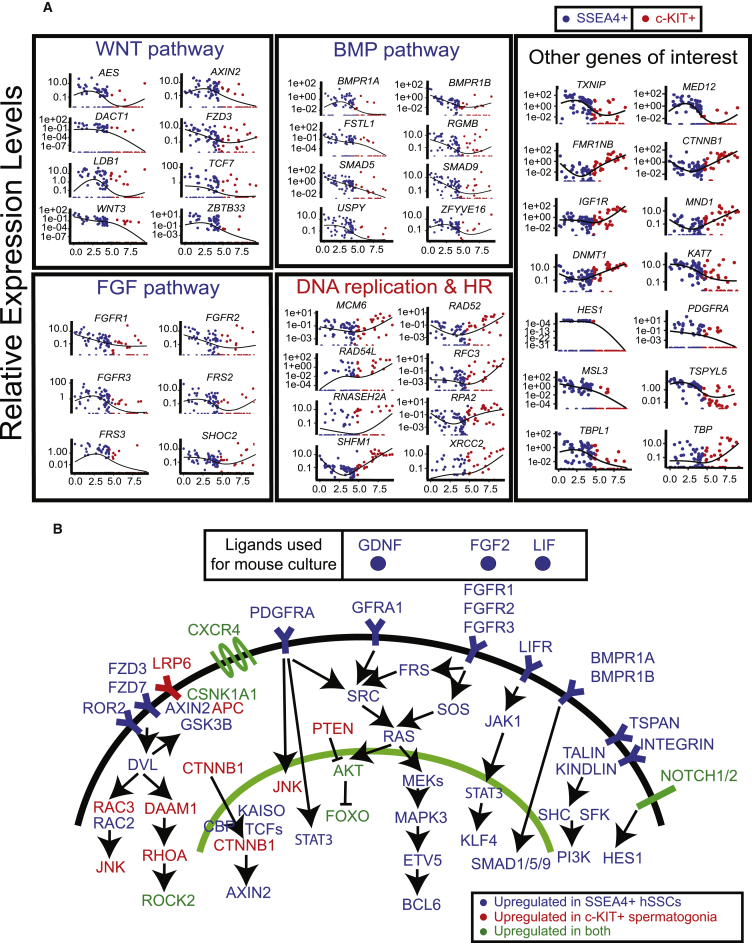
Signaling Pathways Differentially Expressed in SSEA4^+^ hSSCs or c-KIT^+^ Spermatogonia (A) Expression levels of different cell signaling pathway components and other key genes along pseudotime. (B) Schematic summary of signaling pathways singled-out by our RNA-seq analysis, with the ligands currently used in mouse SSCs cultures (outset box). Note: this schematic is based on detection of RNA transcripts and potential signaling activity, not flux measurements. See also Figure S6.

We next investigated in more detail the changes in RNAs encoding signaling factors during spermatogonial transitions, to provide insights into hSSC development and niche-hSSC interaction. Interestingly, WNT, BMP, FGF, LIF, PDGF, GDNF, INTEGRIN/TSPAN, and NOTCH1/HES1 pathway members are more highly expressed in hSSCs compared to c-KIT^+^ spermatogonia (Figure 5A). Notably, ligands for the FGF, GDNF, and LIF signaling pathways are important components of the mouse SSC in vitro culture cocktail (Kanatsu-Shinohara et al., 2003) (Figure 5B). Presently, the roles of WNT, BMP, PDGF, NOTCH and INTEGRIN/TSPAN signaling pathways in SSC culturing are unclear, motivating additional study (see Discussion).

Transcription factors and their chromatin-modifying partners are common targets of signaling pathways and likely mediate the transcriptional changes during hSSC development instructed by germline-niche interactions (Figures 5A and S6). Here, we note that chromatin factors highly expressed in SSEA4^+^ hSSCs included the PRC1 complex (*BMI1*, *PHC1*, *CBX2*), which ubiquitylates the nucleosomal histone H2A, the PR-DUB complex (*BAP1*, *MBD6*), which removes H2A-Ub, and several histone modifiers (*MSL1*, *MSL3*, *EP400*, and *PRMT*-family members). Moreover, the transcription factors, *KLF-*family genes, *SMAD-*family genes (linking to BMP signaling), *TDRD6* (a central component of the chromatoid body in male germ cells), and *TSPYL5*/*6* (Testis-specific protein, Y-linked 4 link 5/6) were also found to be highly expressed in SSEA4^+^ hSSCs. By contrast, we identified mRNAs involved in replication, DNAme (*DNMT1* and *UHRF1*), nonsense mediated decay (*UPF2*), meiosis (*SMC1B*, *MND1*), and DNA replication/recombination expressed at higher levels in c-KIT^+^ spermatogonia. Overall, these findings reveal a large number of factors and pathways that are involved in the regulation of hSSC development and will undoubtedly motivate future functional studies.

### Clustering Analyses Reveal Dynamic Genes and Pathways

**Figure 6:**
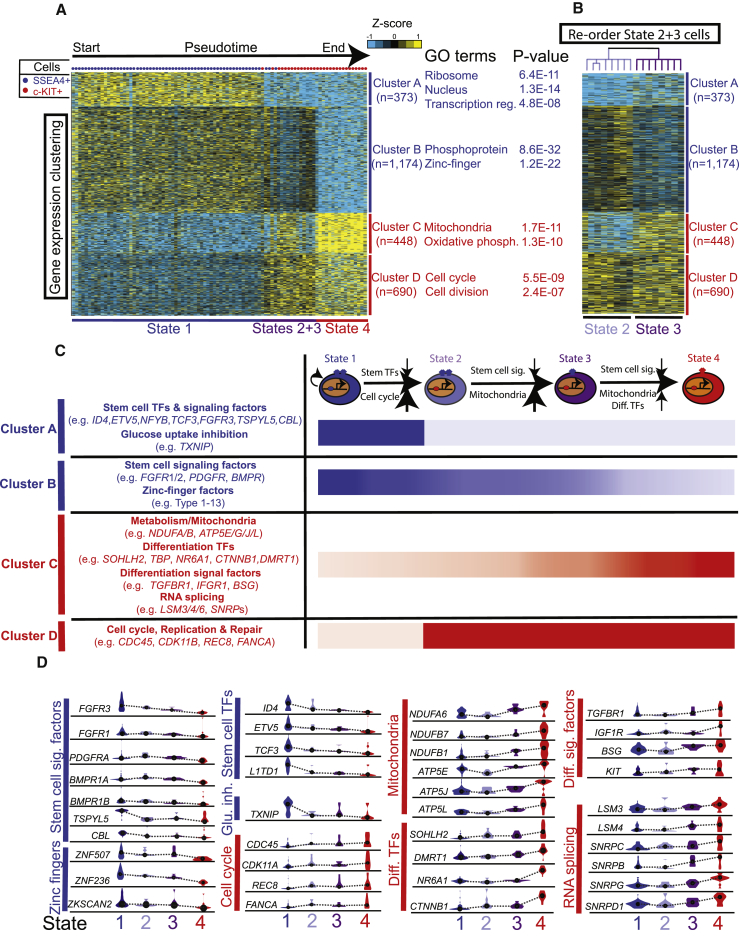
Monocle and Clustering Analyses Reveal Four Cell States For a Figure360 author presentation of Figure 6, see the figure legend at http://dx.doi.org/10.1016/j.stem.2017.09.003. (A) K-means clustering (n = 4) of genes exhibiting differential expression in SSEA4^+^ hSSCs versus c-KIT^+^ spermatogonia. Note: each row represents a gene, and each column represents a single cell, with columns/cells placed in pseudotime order (as defined in Figure 4C). Gene expression levels utilize a *Z* score, which depicts variance from the mean. (B) Hierarchical clustering of state 2 and state 3 cells from Figure 6A. Note: columns (cells) were re-ordered using hierarchical clustering, while genes (rows) were kept in the same order as Figure 6A. These state assignments were then used to refine the identity and trajectory of the minor branches highlighted on the Monocle plot in Figure 4C. (C) Summary schematic of the combinatorial distribution of the four gene expression clusters combine to define four distinct cellular states and proposed dynamic ordering model based on gene identities and pathways. (D) Violin plots of representative genes from each gene cluster, and their relative expression levels in each cellular state. y axis represents *Z* score of expression levels. Here, the mean levels for each state are linked by lines to depict the developmental trajectory. Sig., signaling; TF, transcription factor; Glu. inh., glucose inhibition; Diff., differentiation. Figure360: An Author Presentation of Figure 6

To determine whether coherent cellular “states” exist within the testis, and, if so, how they change during hSSC development, we performed clustering analyses. Here, we placed all single cells in their pseudotime order (compressing the small branches into the larger central branch) and performed k-means clustering on filtered genes (Figure 6A). Interestingly, this approach revealed four distinct gene clusters, hereafter labeled A–D: clusters A and B genes are downregulated along pseudotime development, indicating a correlation with self-renewing hSSCs, whereas clusters C and D genes are upregulated along pseudotime, hence correlating with differentiating spermatogonia. To better understand the biological significance of these four gene clusters, we performed gene ontology and pathway analysis (Figures 6A and 6C), which allowed the grouping of genes into pathways and developmental processes. Cluster A was enriched in RNAs encoding transcription factors known to be associated with mouse SSCs (e.g., *ID4*, *TCF3*, etc.), particular RNA processing factors (LSM and SNRP family), and the central inhibitor of glucose uptake, *TXNIP*; cluster B was enriched in stem cell signaling factors (e.g., FGF and BMP receptors) and zinc finger transcription factors; cluster C was enriched in transcription factors associated with spermatogonial differentiation (e.g., *SOHLH2*, *NR6A1*, *CTNNB1*), signaling receptors (e.g., *IGF1R*, *TGFBR1*), and many mitochondrial factors/regulators (ATP synthase and NADH dehydrogenase subunits and monocarboxylate transport regulation); and cluster D was enriched in genes promoting cell-cycle, replication, and DNA repair factors (e.g., *CDC45, CDK11A, REC8, FANCA*).

### Combining Monocle with Clustering Reveals Four Cellular States

Our examination of how these gene clusters behave and switch their expression in pseudotime suggested the presence of four cellular states. Early pseudotime comprised the vast majority of SSEA4-enriched cells, which displayed a similar single state (termed state 1). On the other hand, cells late in pseudotime were all c-KIT^+^ and displayed a similar state (termed state 4), which exhibited the reciprocal expression pattern of state 1. Visual inspection of the cells at or near the middle branchpoint in Figure 4C, however, showed an intermediate behavior that was highly coherent; all exhibited reversal of cluster A and D expression signatures, but only approximately half of the cells showed reversal of cluster B and C status. To examine these intermediate cells more carefully, we applied hierarchical clustering, which effectively separated these cells into two distinct states, which we termed state 2 and state 3 (Figure 6B). State 2 cells shared the same expression pattern with state 1 cells in clusters B and C but displayed the reciprocal pattern in clusters A and D. State 3 cells then reversed cluster B and C status and thus resembled state 4 cells in all clusters, suggesting that their represent an initial shift toward state 4 status. Remarkably, projection of our state assignments based on this clustering criteria onto the Monocle pseudotime map (Figure 4C, outset below) showed that one of the middle branches is populated almost exclusively by state 2 cells, while the other corresponds to all the cells in state 3.

Thus, by combining Monocle with k-means and hierarchical clustering analysis, we identified four distinct gene clusters, and their differential expression patterns along pseudotime allowed our identification of four distinct cellular states. Further examination of the enriched pathways and genes, along with knowledge of gene/pathway regulation in other stem cell systems, reinforced the order suggested by pseudotime, and prompted us to propose the following dynamic model for hSSC-spermatogonia development (Figures 6C and 6D): self-renewing hSSCs (state 1) are relatively quiescent (with high *TXNIP* inhibiting glucose transport), and have high levels of stem cell-type transcription and signaling factors. They transition into state 2 cells by upregulating cell-cycle and DNA replication/repair factors, while downregulating key stem cell transcription factors and *TXNIP*, allowing glucose import to take place. Transition to state 3 involves the attenuation of stem cell signaling, the upregulation of RNA splicing, and major upregulation of several key mitochondrial activities (that utilize glucose), supporting the production of ATP and lipids required for growth and differentiation. Transition to state 4 involves further reinforcement of this trajectory, via upregulation of transcription factors and signaling pathways that promote spermatogonial differentiation (Figures 6C and 6D). Taken together, a combination of Monocle, clustering, and the examination of impacted genes/pathways reveals an initial logic and dynamic trajectory for hSSC differentiation.

### Validation of scRNA-Seq Data by Immunostaining of Human Seminiferous Tubules

**Figure 7:**
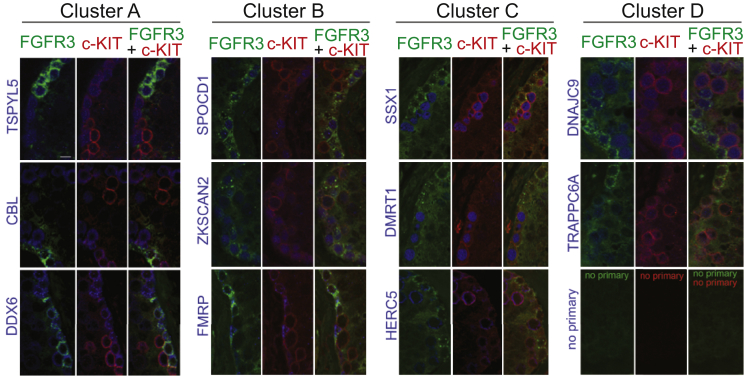
Validation of scRNA-Seq by Immunostaining of Human Seminiferous Tubules Immunolocalization of FGFR3 (cluster A marker used as a surrogate marker to SSEA4, in green), c-KIT (cluster D marker, in red), and 11 different cluster-specific antigens (in blue) on formalin-fixed paraffin embedded (FFPE) sections of human seminiferous tubules. Each antigen (name in blue on the left side) is represented by three panels (left, co-staining with FGFR3; middle, co-staining with c-KIT; right, triple co-IF staining). For clusters A and B, the cluster-specific antigens (blue) are expressed in FGFR3^+^ c-KIT^–^ cells, while, in clusters C and D, the cluster-specific antigens (blue) are expressed in FGFR3^–^ c-KIT^+^ cells. The bottom right tryptic represents the negative (no primary) controls. All pictures are at the same magnification, and the white bar in the top left panel is 10 μm. See also Figure S7.

To further validate the scRNA-seq data and the clustering analysis, we performed triple immunofluorescence (IF) on human testicular sections. To select candidate antigens for validation, we used the online resource of the Human Protein Atlas (http://www.proteinatlas.org/) (Lindskog, 2016, Uhlén et al., 2015) and visualized the protein expression patterns of the 2,685 differentially expressed genes (including 373 genes in cluster A; 1,174 in cluster B; 448 in cluster C and 690 in cluster D) identified by our scRNA-seq clustering analysis. This approach identified ∼250 antigens (42 for cluster A; 98 for cluster B; 51 for cluster C and 62 for cluster D) that were convincingly expressed in cells located along the periphery of the seminiferous tubules. Among these, 11 antibodies (two to three from each cluster), which displayed the best staining quality were selected to perform triple IF stainings in order to determine their pattern of expression in relation to SSEA4^+^ and c-KIT^+^ expressing cells in situ (Figure 7). Given that SSEA4 is a glycolipid carbohydrate epitope for which the available antibody is poorly suited for immunohistochemistry studies (Izadyar et al., 2011), we used antibodies raised to FGFR3, a gene identified by scRNA-seq as a member of cluster A, as a surrogate biomarker to SSEA4. FGFR3 is a well-established human spermatogonial marker (Goriely et al., 2009, Maher et al., 2016), whose expression is restricted to a subpopulation of non-proliferating, non-differentiating hSSCs, typically organized in small clusters of two to four cells, that are all SSEA4^+^ (Figure S7A).

Triple IF with FGFR3, c-KIT and one of the 11 cluster-specific antibodies confirmed that FGFR3 protein expression, like that of SSEA4 (Figure S7A), is largely exclusive from c-KIT (Figures 7 and S7B); protein expression of members of cluster A (TSPYL5, DDX6, CBL) and cluster B (SPOCD1, ZKSCAN2, FMRP) overlapped with FGFR3 but not with c-KIT (Figure 7, left two panels; Figures S7B and S7C), while antibodies directed against members of cluster C (SSX1, DMRT1, HERC5) and cluster D (DNAJC9, TRAPPC6) showed the reciprocal pattern, typically overlapping with a subset of cells most strongly expressing c-KIT (Figure 7, right two panels; Figures S7B–S7D). Taken together, our initial IF validation studies strongly support the trends revealed by the genomics approaches.

## Discussion

Human adult SSCs occupy a critical node in reproductive biology; they need to constantly self-renew to ensure decades of fertility, in balance with commitment to differentiation into spermatogonia, which proliferate and undergo sequential differentiation steps that culminate in the formation of haploid sperm. Although much is known about spermatogonia, their niche, and the germline differentiation process in mice, considerably less is known in humans. By profiling DNAme, chromatin, and transcription in hSSCs, we defined candidate factors and molecular mechanisms underlying the hSSC state—and, through comparisons to c-KIT^+^ spermatogonia, we identified cellular transitions marked by expression changes in transcription factors, signaling, and metabolism that accompany hSSC development, which may also critically inform in vitro culturing.

Prior work in mouse PGCs and SSCs suggests that germline stem cells poise pluripotency factors in a silent but poised state by specific DNAme and chromatin packaging—presumably to enable activation after fertilization (Hammoud et al., 2014). Here, we extend this concept to humans and find *NANOG* and *POU5F1/OCT4*, two of three core pluripotent factors, transcriptionally silent and epigenetically repressed. However, all other pluripotent factors were transcriptionally and epigenetically active. Notably, although *SOX2* is transcriptionally silent, its promoter has open chromatin and is DNA hypomethylated, consistent with its bivalent status in the mouse (Hammoud et al., 2014), prompting future work on bivalency in hSSCs. One attractive candidate for guiding *SOX2* repression is DMRT1, which antagonizes pluripotency in the mouse (Krentz et al., 2009, Takashima et al., 2013). In humans, *DMRT1* mutation is associated with teratoma (Kanetsky et al., 2011, Turnbull et al., 2010), which is linked to aberrant pluripotency pathway expression in the male germline. We found *DMRT1* expression negatively correlated with *SOX2* expression in ESCs, PGCs, and hSSCs, and a *DMRT1* binding motif highly enriched in hSSC-specific ATAC-seq peaks, prompting additional future work. Moreover, our ATAC-seq and transcriptional profiles strongly suggest major roles for CTCF/CTCFL, FOX-family factors, the additional pioneer factor NFYA*/B* and hormone receptors in gene activation in hSSCs. Notably, NFYA/B sites are often located near DMRT1 binding sites (indicating possible co-regulation), and the NFYA/B binding motif is strongly enriched in expressed retrotransposons LTR12C, LTR12D, and LTR12E, which may be utilized to open chromatin for other factors. Finally, the remarkable enrichment of HRE sites in open chromatin, coupled with the high levels of RNA encoding *GR* (data not shown), prompts future studies of glucocorticoid regulation of hSSC function.

Our datasets from MACS-enriched bulk and single-cell populations identified RNAs enriched in SSEA4^+^ or c-KIT^+^ cells. SSEA4-enriched genes included the prominent SSC markers in mice (e.g., *ID4*, *FGFR3*, *SALL4*, *ETV5*), as well as many new candidate hSSC markers, including *TCF7*, *PIWIL2*, *BMPR1A/B*, *L1TD1*, and *TXNIP*. Likewise, we identify a large set of factors, both predicted and novel, more highly expressed in c-KIT^+^ cells, including replication factors (DNMT1 and UHRF1), meiotic factors (SMC1B, MND1), and basic transcription factors (transcription binding protein [TBP]) (Figures 5A and S6).

Although changes in RNA expression do not always lead to protein dynamics, the co-immunostaining results has allowed us to confirm, via direct visualization, that changes in gene expression uncovered at the single-cell level often translate into discrete protein expression within specific sub-populations of cells located at the periphery of the seminiferous tubules. Although we analyzed only 11 antigens, they exhibited distinct expression patterns, underscoring the considerable phenotypic heterogeneity that exists within the FGFR3^+^/SSEA4^+^ and c-KIT^+^ cell populations. Further work detailing the relative protein expression patterns encoded by the differentially expressed genes identified here will aim to delineate sub-populations of hSSCs with differential developmental potentials. Here, further work with larger numbers of single cells (via scRNA-seq or FACS) may reveal additional cell states that correspond to these sub-populations, or instead, may reveal cells with similar gene expression states with alternative post-transcriptional/proteomic sub-states. Importantly, the data generated by the scRNA-seq clustering analysis provide a unique opportunity to identify novel diagnostic biomarkers that can be used to further stratify discrete immuno-phenotypes and developmental sub-states. Furthermore, our studies may be useful in identifying a sub-population within the SSEA4^+^ population that possesses more “stem-like” properties, which could be tested in future functional assays.

Understanding germ cell developmental/metabolic states and how they might be determined by signaling from the niche is likely an important prerequisite for successful culturing of hSSCs. Whereas mouse SSCs can be cultured long term, hSSCs quickly lose germ cell identity in culture—thus preventing their use in therapeutic applications (Medrano et al., 2016, Zheng et al., 2014)—for example, to restore fertility to prepubertal cancer survivors after chemo- or radiation therapy (Valli et al., 2014b). Here, we reveal differences in the transcription levels of components of particular signaling pathways in self-renewing SSEA4^+^ hSSCs compared to differentiating c-KIT^+^ spermatogonia that may inform culturing, but note that pathway flux/activity was not directly measured. Among them, LIF, FGF, and GDNF/GFRA1 pathways are already of known importance for culturing mouse SSCs (Kanatsu-Shinohara et al., 2003). More importantly, our work has revealed multiple additional pathways not previously explored in SSC culturing, including NOTCH, PDGF, BMP, and TSPAN/INTEGRIN pathways. For example, the HES1 repressor is important for neuronal stem cell maintenance, with precocious differentiation in its absence (Kabos et al., 2002). Thus, hSSCs may rely on an active NOTCH pathway for self renewal. Furthermore, we observe that WNT signaling is transcriptionally regulated differently in SSEA4^+^ hSSCs versus c-KIT^+^ cells. Although the role of WNT signaling in hSSCs remains to be established, activation of the WNT pathway promotes the differentiation of mouse SSCs (Takase and Nusse, 2016).

Remarkably, we found that by combining Monocle and clustering analyses (k-means and hierarchical) to the single-cell datasets, four distinct cellular states emerged. This partitioning, and the remarkable degree to which the gene clusters “flip” expression profiles to define subsequent states, strongly suggests the presence of feedback loops: positive feedback loops within clusters, and negative feedback loops between clusters, which we suggest may provide coherence to the developmental transitions and allow robustness during development. Moreover, the grouping of the genes within each cluster, along with pathway analyses and knowledge from other stem cell systems (e.g., hematopoietic and neuronal stem cells), has allowed us to propose a dynamic model for human spermatogonial development (Ito and Suda, 2014). Intuitively, self-renewing hSSCs (state 1) should be relatively quiescent (with high TXNIP levels inhibiting glucose transport) and should be enriched in stem cell-type transcription and signaling factors. Transition to state 2 involves upregulating cell-cycle and DNA replication/repair factors and downregulating stem cell-type transcription factors—with strong *TXNIP* repression enabling efficient glucose import, possibly to fuel the subsequent state. Transition to state 3 involves the downregulation of stem cell signaling factors and the strong upregulation of RNA splicing/processing factors and central mitochondrial activities (e.g., ATP synthase and NADH dehydrogenase, and also monocarboxylate transport [via *BSG*]) that facilitates ATP and lipid production. Transition to state 4 reinforces this trajectory, leading to the upregulation of key transcription factors and signaling pathways that promote spermatogonial differentiation (Figures 6C and 6D). Thus, our study identifies four cellular states and revealed a logical developmental trajectory that accounts for the transition of hSSCs from quiescence to proliferation and differentiation. We hope this model will provide a general framework to generate hypothesis-driven experiments to further assess the development of this important and unique stem cell population.

## STAR★Methods

### Key Resources Table

**Table:** 

REAGENT or RESOURCE	SOURCE	IDENTIFIER
**Antibodies**		

Mouse monoclonal anti-SSEA4; dilution: 1:25 - 1:100	Cell Signaling Technology	cat# 4755, clone MC813, RRID: AB_1264259
Goat polyclonal anti-c-KIT (CD117); Dilution: 1:25 - 1:100	R&D Systems	cat# AF332, RRID: AB_355302
Rabbit polyclonal anti-cFGFR3; dilution: 1:500	Santa Cruz Biotechnology	cat# sc390423, clone C-15, RRID: AB_631511
Mouse monoclonal anti-nFGFR3; dilution: 1:500- 1:1000	Santa Cruz Biotechnology	cat# sc1312, clone B-9, RRID: AB_627596
Rabbit polyclonal anti-DDX6; dilution: 1:500	Atlas Antibodies	cat# HPA024201, RRID: AB_10603562
Rabbit polyclonal anti-TSPYL5; dilution: 1:5000	Atlas Antibodies	cat# HPA031347, RRID: AB_10601454
Rabbit polyclonal anti-CBL; dilution: 1:500	Atlas Antibodies	cat# HPA027956, RRID: AB_10601094
Rabbit polyclonal anti-SPOCD1; dilution: 1:300	Atlas Antibodies	cat# HPA031715, RRID: AB_2674008
Rabbit polyclonal anti-ZKSCAN2; dilution: 1:250	Atlas Antibodies	cat# HPA049141, RRID: AB_2680652
Rabbit polyclonal anti-FMRP; dilution: 1:700	Abcam	cat# ab27455, RRID: AB_732400
Rabbit polyclonal anti-HERC5; dilution: 1:400	Atlas Antibodies	cat# HPA043929, RRID: AB_10962492
Rabbit polyclonal anti-SSX1; dilution: 1:300	Atlas Antibodies	cat# HPA045683, RRID: AB_2679418
Rabbit polyclonal anti-DMRT1; dilution: 1:1000	Atlas Antibodies	cat# HPA027850, RRID: AB_10600868
Rabbit polyclonal anti-TRAPPC6A; dilution: 1:200	Atlas Antibodies	cat# HPA043043, RRID: AB_10794650
Rabbit polyclonal anti-DNAJC9; dilution: 1:200	Atlas Antibodies	cat# HPA035215, RRID: AB_10603663
Goat Polyclonal anti-GFRA1; dilution: 1:400	R&D systems	cat# AF560, RRID: AB_2110307
Donkey-anti Goat Alexa 594; dilution: 1:200	Thermo Fisher Scientific	cat# A-11058, RRID:AB_2534105
Donkey anti-Mouse IgG (H+L) Highly Cross-Adsorbed Secondary Antibody, Alexa Fluor 488	Thermo Fisher Scientific	cat# A21202, RRID:AB_141607
Donkey anti-Goat IgG (H+L) Highly Cross-Absorbed Secondary Antibody, Alexa Fluor 555	Thermo Fisher Scientific	cat# A21432, RRID:AB_141788
Donkey anti-Rabbit IgG (H+L) Highly Cross-Adsorbed Secondary Antibody, Alexa Fluor 647	Thermo Fisher Scientific	cat# A31573, RRID:AB_2536183

**Biological Samples**		

Human testis samples for genomics	University of Utah Andrology Laboratory	NA
Human testis samples for immunofluorescence	Department of Cellular Pathology/Oxford Centre for Histopathology Research, John Radcliffe Hospital, Oxford	NA

**Critical Commercial Assays**		

SSEA4 MicroBeads	Miltenyi Biotec	cat# 130-097-855
c-KIT MicroBeads	Miltenyi Biotec	cat# 130-098-571

**Deposited Data**		

Whole Genome Bisulfite Sequencing	This paper	GEO: GSE92280
ATAC-seq	This paper	GEO: GSE92280
Bulk RNA-seq	This paper	GEO: GSE92280
Single Cell RNA-seq	This paper	GEO: GSE92280
Human PGC and Liver DNAme data	Guo et al., 2015	GEO: GSE63818
ICM and FC DNAme data	Guo et al., 2014a	GEO: GSE49828
Oocyte DNAme data	Okae et al., 2014	JGAS00000000006
ESC DNAme data	Gifford et al., 2013	GEO: GSM1112834
Sperm DNAme data	Hammoud et al., 2009	GEO: GSE15594
ESC and PGC RNA-seq	Gkountela et al., 2015	GEO: GSE63392
Human oocyte and early embryo RNA-seq	Hendrickson et al., 2017	GEO: GSE85632
The Human Protein Atlas	Lindskog, 2016	RRID: SCR_006710; http://www.proteinatlas.org

**Software and Algorithms**		

Monocle (v 1.2.0)	Trapnell et al., 2014	http://cole-trapnell-lab.github.io/monocle-release/
Useq Package (v 8.8.8)	Nix et al., 2010	http://useq.sourceforge.net
GO (David 6.7)	Huang et al., 2009	https://david-d.ncifcrf.gov
Homer (v 4.8.3)	Heinz et al., 2010	http://homer.ucsd.edu/homer/
Novoalign (v 2.8)	N/A	http://www.novocraft.com
Cluster 3.0	N/A	http://bonsai.hgc.jp/∼mdehoon/software/cluster/software.htm
deepTools (v 3)	Ramírez et al., 2014	https://deeptools.github.io
Rtsne (v 0.10)	N/A	R package
pheatmap (v 1.0.8)	N/A	R package
Samtools (v 1.4)	Li et al., 2009	http://samtools.sourceforge.net/
Macs2 (v2.1.120160309)	Zhang et al., 2008	https://github.com/taoliu/MACS
FactoMineR	N/A	http://factominer.free.fr
DESeq2	Love et al., 2014	http://bioconductor.org/packages/release/bioc/html/DESeq2.html
Bio-ToolBox (v1.40)	N/A	https://github.com/tjparnell/biotoolbox
FlowJo (v10.1)	FlowJo	https://www.flowjo.com/solutions/flowjo

### Contact for Reagent and Resource Sharing

Further information and requests for reagents and resources should be directed to and will be fulfilled by the Lead Contact, Bradley R. Cairns (brad.cairns@hci.utah.edu)

### Experimental Model and Subject Details

Healthy adult human testis samples for genomics profilings were obtained from five men experiencing idiopathic pain, not involving cancer or major inflammation, through the University of Utah Andrology laboratory consented for research (IRB approved protocol #00075836: understanding the transcriptional and epigenetic dynamics in human spermatogonial stem cell self-renewal, proliferation and differentiation).

The majority of samples used for immunofluorescence were prepared from formalin-fixed paraffin embedded (FFPE) non-malignant testes removed from anonymized patients for reasons of coincidental pathology and were acquired from the Department of Cellular Pathology/Oxford Centre for Histopathology Research (OCHRe), John Radcliffe Hospital, Oxford, UK, as previously described (Lim et al., 2012). All patients had given informed written consent for research use and ethical approval was provided by the Oxfordshire Research Ethics Committee A (C03.076: Receptor tyrosine kinases and germ cell development).

### Method Details

#### Human Testis Samples Preparation for Genomics Profilings

For genomics profilings, collected testes were transported to the research laboratory on ice in Hank’s Balanced Salt Solution (HBSS; GIBCO cat# 24020117) within 1 hr. Large tissues were divided into smaller sizes (around 500mg – 1g each) using scissors. Single testicular cells were obtained using two-step enzymatic digestion described in (Valli et al., 2014a). Briefly, testicular tissue was digested with collagenase type IV (Sigma Aldrich cat# C5138-500MG) for 5 min at 37° C on the shaker (250 rpm), then shaken vigorously and incubated for another 3 min. The tubules were sedimented by centrifugation at 200 g for 5 min and washed with HBSS before digestion with 4.5 mL 0.25% trypsin/ethylenediaminetetraacetic acid (EDTA; Invitrogen cat# 25300054) and 4 ku DNase I (Sigma-Aldrich cat# D4527-500ku). The suspension was triturated vigorously three to five times and incubated at 37° C for 5 min. The process was repeated in 5 min increments for up to 15 min total. The digestion was stopped by adding 10% fetal bovine serum (FBS; GIBCO cat# 10082147) and the cells were size-filtered through 70 μm (Fisher Scientific cat# 08-771-2) and 40 μm strainers (Fisher Scientific cat# 08-771-1). The cells were pelleted by centrifugation at 600 g for 15 min.

#### Human Spermatogonia Isolation using MACS

SSEA4+ or c-KIT+ cells were enriched using magnetic activated cell sorting (MACS) protocols (Miltenyi Biotec). For SSEA4 enrichment, single testicular cell suspensions were incubated with anti-SSEA4 microbeads (Miltenyi Biotec cat# 130-097-855) at 4°C. For KIT+ cells selection, single testicular cell suspensions were incubated with anti-cKIT microbeas (Miltenyi Biotec cat# 130-098-571) at 4°C. Following microbead binding, cells were re-suspended in autoMACS running buffer (Miltenyi Biotec cat# 130-091-221) and ran through LS columns (Miltenyi Biotec cat# 130-042-401) placed in a magnetic field. Columns were rinsed three times with buffer in autoMACS running buffer (Miltenyi Biotec cat# 130-091-221) before being removed from the magnetic field. MACS running/separation buffer (Miltenyi Biotec cat# 130-091-221) was then applied to the column before magnetically-labeled cells were flushed out by firmly pushing the plunger into the column. Cells were then centrifuged and re-suspended to a desired concentration. Starting with half a testicle, after dissociation and filtering with strainers, a total number of ∼26 million testicular cells were recovered. SSEA4 MACS sorting, yielded ∼0.26 million cells; while KIT MACS sorting yielded ∼0.2 million cells.

#### FACS Analysis

Cells were analyzed by flow cytometry using Fortessa Analyzer. After incubation with anti-SSEA4 microbeads (Miltenyi Biotec cat# 130-097-855), cells were stained using labeling check reagent-FITC (Miltenyi Biotec cat# 130-099-136) and DAPI (Life Technologies cat# P36931) following manufacturer’s instructions. Unstained cells were used as control. FACS data were analyzed using FlowJo software (Ashland).

#### Immunocytochemistry on Sorted Cells

Immunofluorescence analysis of sorted cells was performed as described below. Briefly, cells were prepared by adhesion to poly-D-lysine coated coverslips (BD Biosciences cat# 354086). Coverslips were washed in 1 × PBS and fixed in 4% paraformaldehyde /1 x PBS for 10 min at room temperature (Electron Microscopy Sciences Hatfield, PA USA, 15710). Following a 1 x PBS wash, the cells were permeabilized with 1 x PBS + 0.1% Triton X-100 at room temperature for 10 min, rinsed in 1 x PBS and blocked in 3% BSA /1 × PBS for 1 hr. Goat anti-GFRA1 (1:400; R&D Systems AF560) primary antibody was diluted into 3% BSA/1 x PBS and incubated overnight at 4°C with no rocking in a hybridization chamber. Coverslips were then washed 3 times with 1 × PBS for 15 min before incubation of the secondary antibody (Donkey-anti Goat Alexa 594 (Thermo Fisher Scientific cat# A-11058)) for 1 hr at room temperature. Finally, cells were washed 3 times in 1 x PBS for 15 min and mounted onto glass slides with ProLong® Gold Antifade mounting reagent containing DAPI (Life Technologies, cat# P36931). Cells were imaged on Nikon A1 Ti-E inverted microscope equipped with Four Photo Multipliers Tube (PMT) detector unit. Images were taken utilizing 405nM (for DAPI detection) and 561nm Sapphire diode laser (for RFP detection) under a 60x oil immersion objective. Z sections were acquired for a plane of cells at 0.5 μm steps.

#### Single Cell Transcriptome Sequencing

Isolated SSEA4+ or c-KIT+ cells were diluted to 15,000-20,000 cells/μl in cold PBS and loaded into 5-10 μm integrated fluidic circuits (IFCs) using Fluidigm C1 instrument. Single cells captured in IFCs were scored under microscope. Only cells with normal morphology were selected for sequencing. Single cells in IFCs were lysed and total RNA was harvested for polyadenylation selection, reverse transcription and PCR amplification. Library constructions were performed following guidelines of the Fluidigm Library preparation with Nextera XT protocol and sequenced on a 50-cycle single end run on an Illumina HiSeq 2500 instrument.

#### RNA Extraction and Bulk Transcriptome Sequencing

RNA was extracted from pooled SSEA4+ or KIT+ cells using AllPrep RNA/DNA/Protein Mini Kit (QIAGEN cat# 80004). Total RNA was then subjected to RiboZero Gold (Illumina cat# MRZG126) to substantially deplete cytoplasmic and mitochondrial rRNA. Standard RNA sequencing libraries were prepared as described using the Illumina TruSeq Stranded Total RNA Kit with Ribo-Zero Gold and sequenced on a 50-cycle single end run on an Illumina HiSeq 2500 instrument.

#### Genomic DNA Extraction and Whole Genome Bisulfite Sequencing

Genomic DNA was extracted from pooled SSEA4+ cells using AllPrep RNA/DNA/Protein Mini Kit (QIAGEN cat# 80004). Library construction was performed using the EpiGnome Methyl-Seq Kit (Epicenter EGMsK89312) as described below. Briefly, genomic DNA (50-100 ng) was denatured and bisulfite converted using the EZ DNA Methylation-Gold Kit (Zymo Research cat# D5005) in a reaction containing 0.5 ng of unmethylated lambda DNA (Promega cat# D1521) as a control. Following purification, the bisulfite converted DNA was hybridized with oligonucleotides consisting of random hexamers linked to Illumina P5 adaptor sequence and strand replication was accomplished using EpiGnome polymerase. Double-stranded DNA was heat-denatured to enable ligation of the EpiGnome Terminal Tagging Oligo which adds Illumina P7 adaptor sequence to the 3′ end of the replicated strand. Adaptor-ligated DNA molecules were enriched by 10 cycles of PCR and the amplified library was subsequently purified using Agencourt AMPure XP beads (Beckman Coulter cat# A63881). The concentration of the library was measured using the Qubit dsDNA HS Assay (Invitrogen cat# Q32854) and an aliquot of the library was run on an Agilent 2200 Tape Station using a D1000 (cat# 5067-5582 and 5067-5583) or a High Sensitivity D10000 (cat# 5067-5584 and 5067-5587) assay to define the size distribution of the sequencing library. Libraries were diluted to a concentration of approximately 10 nM and quantitative PCR was performed using the Kapa Library Quant Kit (Kapa Biosystems cat# KK4824) to calculate the proportion of adaptor-ligated DNA molecules. The concentration was further adjusted following qPCR to prepare the library for Illumina sequencing. Libraries were then sequenced on a 125-cycle paired-end run on an Illumina HiSeq 2500 instrument.

#### ATAC-seq Library Preparation and Sequencing

The ATAC-seq libraries were prepared as previously described (Buenrostro et al., 2013) on ∼30k sorted SSEA4+ SSCs or cultured ESCs. Briefly, collected cells were lysed in cold lysis buffer (10 mM Tris-HCl, pH 7.4, 10 mM NaCl, 3 mM MgCl2 and 0.1% IGEPAL CA-630) and the nuclei were pelleted and resuspended in Transposase buffer. The Tn5 enzyme was made in-house (Picelli et al., 2014) and the transposition reaction was carried out for 30 min at 37°C. Following purification, the Nextera libraries were amplified for 12 cycles using the NEBnext PCR master mix (NEB cat# M0541L) and purified using the Agencourt AMPure XP – PCR Purication (Beckman Coulter cat# A63881). All libraries were sequenced on a 125-cycle paired-end run on an Illumina HiSeq 2500 instrument.

#### Immunostaining of Testis Tissues

Most of the triple immunofluorescence stainings were performed on 5μm formalin-fixed paraffin embedded (FFPE) sections following deparaffinisation, rehydratation and heat-mediated antigen retrieval in 10mM Sodium citrate buffer solution (pH 6). In order to block non-specific binding, sections were treated with Superblock (PBS) Blocking Buffer (Thermo Fisher Scientific cat# 37515) for 30 min. They were then incubated overnight at 4°C with a mix of three diluted antibodies (nFGFR3 (Cluster A marker, mouse monoclonal), c-KIT (Cluster D marker, goat polyclonal) and a third rabbit polyclonal antibody (for antibodies details and dilutions, see the Key Resources Table above and Table S4). Subsequently, antigen detection was conducted using the appropriate combination of Alexa Fluor 488, 555 and 647 secondary antibodies (all 1:500; Thermo Fisher Scientific cat# A21202, cat# A21432, cat# A31573 respectively) for 2 hr at room temperature in the dark. All primary/secondary antibodies were diluted in SignalBoost Immunoreaction Enhancer Kit (Calbiochem cat# 407207-1KIT). After three washes in PBS, DAPI (4’,6-Diamidine-2-phenylindole dihydrochloride) (Roche cat# 10 236 276 001) at a dilution 1:2000 was used for nuclear visualization. Specificity of the antibody staining was confirmed using the same protocol but with omission of primary antibodies. Following multiple washes in PBS, slides were preserved using Vectashield mounting medium for fluorescence (Vector Laboratories cat# H-1000). Images were obtained under 25x objective (LD LCI PA 25x/0.8 DIC WD = 0.57 mm Imm Corr (UV)VIS-IR (Multi-Immersion (Oil, glycerine), water) with a Zeiss LSM 780 Upright Multi-Photon Confocal Microscope and analyzed using ImageJ software.

Because of the extracellular localization of the SSEA4 epitope, treatment of tissues with detergents or alcohols had to be omitted and therefore the triple SSEA4/cFGFR3/c-KIT immunostaining was performed on 8μm OCT-embedded cryosections. Slides were thawed and rinsed three times for 15 min in PBS at room temperature and the same procedure as for the FPPE material from the blocking of non-specific binding reaction onward.

### Quantifications and Statistical Analysis

#### Whole Genome Bisulfite Sequencing Analysis

WGBS analysis pipeline was modified from our previous work (Potok et al., 2013). Briefly, fastq files were first aligned to human hg19 genome by novoalign aligner, then processed and analyzed by USeq package (http://useq.sourceforge.net) (Nix et al., 2010). Customized analysis was performed using Bio-ToolBox (https://github.com/tjparnell/biotoolbox) and R. All applications used are open source. Detailed analysis procedures are listed below: WGBS fastq files were aligned from Illumina Fastq files to human hg19 genome using Novocraft’s novoalign aligner (http://www.novocraft.com) in ‘bisulfite’ mode with the following parameters: -o SAM –h 120 –t 240 –b 2 –R 3. An in silico chrLambda sequence was used to align the fully methylated lambda sequence that was spiked into the samples in order to measure the bisulfite conversion efficiency. Bisulfite alignment and parsing was done using NovoalignBisulfiteParser (http://useq.sourceforge.net/cmdLnMenus.html#NovoalignBisulfiteParser) application and the point data was then parsed into mCG context using the ParsePointDataContexts (http://useq.sourceforge.net/cmdLnMenus.html#ParsePointDataContexts) application. Then BisStat (http://useq.sourceforge.net/cmdLnMenus.html#BisStat) was used to calculate per base fraction methylation scores for bases with five or more reads from both strands and generate tracks in useq format. Those useq files can be converted into bigwig files for visualization in IGV using the USeq2UCSCBig (http://useq.sourceforge.net/cmdLnMenus.html#USeq2UCSCBig) application. For further analysis, those bigwig files can be converted into bedgraph format using UCSC bigWigToBedGraph (http://hgdownload.cse.ucsc.edu/admin/exe/) application. The allelic methylation detector (AMD; http://useq.sourceforge.net/cmdLnMenus.html#AllelicMethylationDetector) was used to test whether methylation dynamic in certain regions is caused by bimodal distribution. CpG methylation levels of two biological samples (in bedgraph format) were merged by genomic coordinates, and Person correlation coefficient (r) between them were calculated. Only CpG that were captured in both samples were taken into account. With a high correlation coefficient (r = 0.844), two SAM files were merged using the MergeSamFiles application in Picard (http://broadinstitute.github.io/picard/command-line-overview.html#MergeSamFiles). Merged SAM file was then processed following the same procedures described above.

#### Comparison of DNA Methylation Between Different Tissue Types

Human DNA methylation datasets of different human cell were downloaded from published datasets: human PGC and Liver methylation data from (Guo et al., 2015); Inner Cell Mass and Frontal Cortex (FC) methylation data from (Guo et al., 2014a); Oocyte methylation data from (Okae et al., 2014); ESC methylation data from (Gifford et al., 2013); human sperm methylation data from (Hammoud et al., 2009). Those datasets and human germline stem cell methylation data were merged by their genomic coordinates using “merge” function in R. Hierarchical clustering and heatmap display were performed using “pheatmap” package in R. Correlation analysis was carried out by calculating Pearson correlation coefficient between different tissue types. The getdataset application from Bio-ToolBox was used to calculate average DNA methylation levels in given genomic regions.

#### Repetitive Element Expression Analysis

Genomic coordinate table of repetitive elements was downloaded from UCSC Genome Bioinformatics (hg19) (https://genome.ucsc.edu/cgi-bin/hgTables). RNA-seq data of human oocyte and early embryos are from (Hendrickson et al., 2017). Reads that map to repetitive elements were collected using the getdataset application in Bio-ToolBox. Reads were then normalized by total length of repetitive element (sum of length of all genomic loci) and total mapped reads.

#### ATAC-seq Analysis

Customized analysis was performed using Bio-ToolBox (https://github.com/tjparnell/biotoolbox, v1.40) BedTools (http://bedtools.readthedocs.io/en/latest/content/tools/makewindows.html, v2.17.0) and R. SAM alignments were generated from Illumina Fastq files aligned to human hg19 genome using Novocraft’s novoalign aligner (http://www.novocraft.com) with the following parameters: -o SAM –r ALL 50. Peak calling was performed using macs2 (https://github.com/taoliu/MACS, v2.1.1.20160309) using the following settings: -g 2.7e9–call-summit –f BAMPE –nomodel –B –SPMR –extsize 200. Generated bedgraph file was then transformed to bw format using UCSC bedGraphToBigWig application (v4). Heatmap clustering of ATAC-seq were carried out using deepTools (v3). bw files from ATAC-seq were first normalized. Matrix was generated using computeMatrix application using the following parameters: computeMatrix -S input_1.bw input_2.bw -R peaks.bed–outFileName out.matrix–referencePoint center -a 5000 -b 5000 -bs 100–sortRegions descend–maxThreshold 1. The peaks.bed was genereated by combining peaks from ESCs and SSCs. plotHeatmap application was then used to plot heatmap: plotHeatmap -m out.matrix–kmeans n–dpi 1000–outFileNameMatrix nout.tab –outFileSortedRegions out.bed. Motif finding analysis was carried out using findMotifGenome.pl application (v4.8.3, homer).

#### Bulk RNA Sequencing Analysis

SAM alignments were generated from Illumina Fastq files aligned to human hg19 genome using Novocraft’s novoalign aligner (http://www.novocraft.com, v2.8) with the following parameters: -o SAM –r ALL 50. Counts and RPKMs for Ensemble genes were determined with DefinedRegionDifferentialSeq (DRDS: http://useq.sourceforge.net/cmdLnMenus.html#DefinedRegionDifferentialSeq, v8.8.8, Useq) application. ESC and PGC RNaseq data were obtained from (Gkountela et al., 2015) and reprocessed as described above. The gene expression level of ensemble genes was log scaled, and Pearson correlation coefficient (r) between technical replicates were calculated using customized R script. To perform principal component (PCA) analysis, gene expression were transformed by log(1+FPKMs). Then PCA was conducted using FactoMineR package (http://factominer.free.fr). Differential gene expression analysis was performed using R bioconductor, DESeq2 package (Love et al., 2014). This method took in raw data counts, which were used to fit a negative binomial distribution model, and the false discovery rate (FDR) was used to correct for multiple testing errors. Only the genes with significant p values and FDR less than 0.05 were considered to be differentially expressed. David bioinformatics resources 6.7 (https://david.ncifcrf.gov) were used for gene ontology enrichment analysis.

#### Single Cell RNA Sequencing Analysis

SAM alignments were generated from Illumina Fastq files aligned to human hg19 genome using Novocraft’s novoalign aligner (http://www.novocraft.com) with the following parameters: -o SAM –r ALL 50. Raw data counts were collected using DefinedRegionDifferentialSeq (DRDS: http://useq.sourceforge.net/cmdLnMenus.html#DefinedRegionDifferentialSeq) application using the following parameters: -m -t. RPKM (read per kilobase of transcript per million mapped reads) was calculated using customized R scripts. Single cell samples with more than 2 million reads were retained for further analysis. In order to eliminate sparseness, single cells that expressed at least 3000 genes (RPKM > 0.5) and genes that expressed in at least 20 single cells (RPKM > 0.5) were retained for further analysis, resulting in 92 single cells and 9000 genes. Differential gene expression analysis was carried using R bioconductor, Monocle package (v1.0) (Trapnell et al., 2014), which yielded 2685 genes (FDR < 0.1). Those 2685 differential expressed genes and 92 single cells were then used for further analysis. t-SNE analysis of single cell transcriptome was performed using Rtsne package (v0.1-3). Analysis of transcriptome dynamic along pseudotime was performed using R bioconductor, Monocle package (v1.0) (Trapnell et al., 2014). K-means clustering analysis was performed using Cluster 3.0 (http://bonsai.hgc.jp/∼mdehoon/software/cluster/software.htm) application. In this analysis, each column represented a single cell sample and each row represented a gene. Based on Monocle analysis, single cells (columns) were aligned in the order of pseudo development, and genes that were discovered to be differentially expressed by Monocle (rows) were clustered using k-means clustering using Cluster 3.0.

### Data and Software Availability

The accession number for the whole-genome bisulfite sequencing, ATAC-seq, bulk and single-cell RNA-seq data reported in this paper is GEO: GSE92280.

## Author Contributions

Overall Design, B.R.C. and J.G.; Acquisition of Samples, J.M.H. and D.T.C.; Sample Processing, J.G. and C.Y.; Detailed Molecular and Genomics Approaches, J.G., E.J.G., and C.L.W.; Sequencing Analysis, J.G. and P.J.M.; Data Analysis and Figures 1, 2, 3, 4, 5, and 6, J.G. and B.R.C.; Immunostainings and Figure 7, A.G., H.M., and G.J.M.; HPA Antibodies, C.L. J.G., A.G., and B.R.C. wrote the manuscript with comments from all authors.

## Supporting Citations

The following references appear in the Supplemental Information: Pastor et al. (2016).

## Supplemental Information

Document S1. Figures S1–S7

Table S1. Genomic Summary of Bulk and Single-Cell Sequencing, Related to Figure 1 and STAR Methods

Table S2. Different Clusters of ATAC-Seq Peaks, Related to Figure 2

Table S3. Summary of Single-Cell Sequencing Analysis, Related to Figures 4, 5, and 6

Table S4. Antibodies Used for Immunostaining Validation on Testicular Sections, Related to Figure 7

Document S2. Article plus Supplemental Information
