# Supplementary material for: Chromatin and Single-Cell RNA-Seq Profiling Reveal Dynamic Signaling and Metabolic Transitions during Human Spermatogonial Stem Cell Development
Source: Cell Stem Cell. 2017 Oct 5;21(4):533–546.e6. doi: 10.1016/j.stem.2017.09.003 (PMC5832720; doi:10.1016/j.stem.2017.09.003)
Supplement: Document S1. Figures S1–S7 [file mmc1.pdf]

Cell Stem Cell, Volume *21*

Supplemental Information

Chromatin and Single-Cell RNA-Seq Profiling Reveal Dynamic Signaling and Metabolic Transitions during Human Spermatogonial Stem Cell Development

Jingtao Guo, Edward J. Grow, Chongil Yi, Hana Mlcochova, Geoffrey J. Maher, Cecilia Lindskog, Patrick J. Murphy, Candice L. Wike, Douglas T. Carrell, Anne Goriely, James M. Hotaling, and Bradley R. Cairns

Supplemental Data

Figure S1. Genomic Profiling of hSSCs. Related to Figure 1.

Figure S2. DNAme Dynamics in Different Tissues at Known Imprinted Sites. Related to Figure 1.

Figure S3. Comparison of ATAC-seq Signals Between hSSCs and ESCs. Related to Figure 2.

Figure S4. Expression, DNAme and ATAC-seq Signals at Repetitive Elements. Related to Figure 2.

Figure S5. Profiling of Transcriptome (RNA-seq) in hSSCs. Related to Figures 3 and 4.

Figure S6. Expression of Representative Selected Key Genes along 'Pseudotime' in SSEA4+ and c-KIT+ single cells. Related to Figures 4-6.

Figure S7. Direct Visualisation of Protein Expression by Immunofluorescence Validate Predictions from the Genomics Data Clustering. Related to Figure 7.

Table S1. Genomic Summary of Bulk and Single Cell Sequencing. Related to Figure 1 and STAR methods.

Table S2. Different Clusters of ATAC-seq Peaks. Related to Figure 2.

Table S3. Summary of Single Cell Sequencing Analysis. Related to Figures 4-6.

Table S4. Antibodies Used for Immunostaining Validation on Testicular Sections. Related to Figure 7.

Figure S1

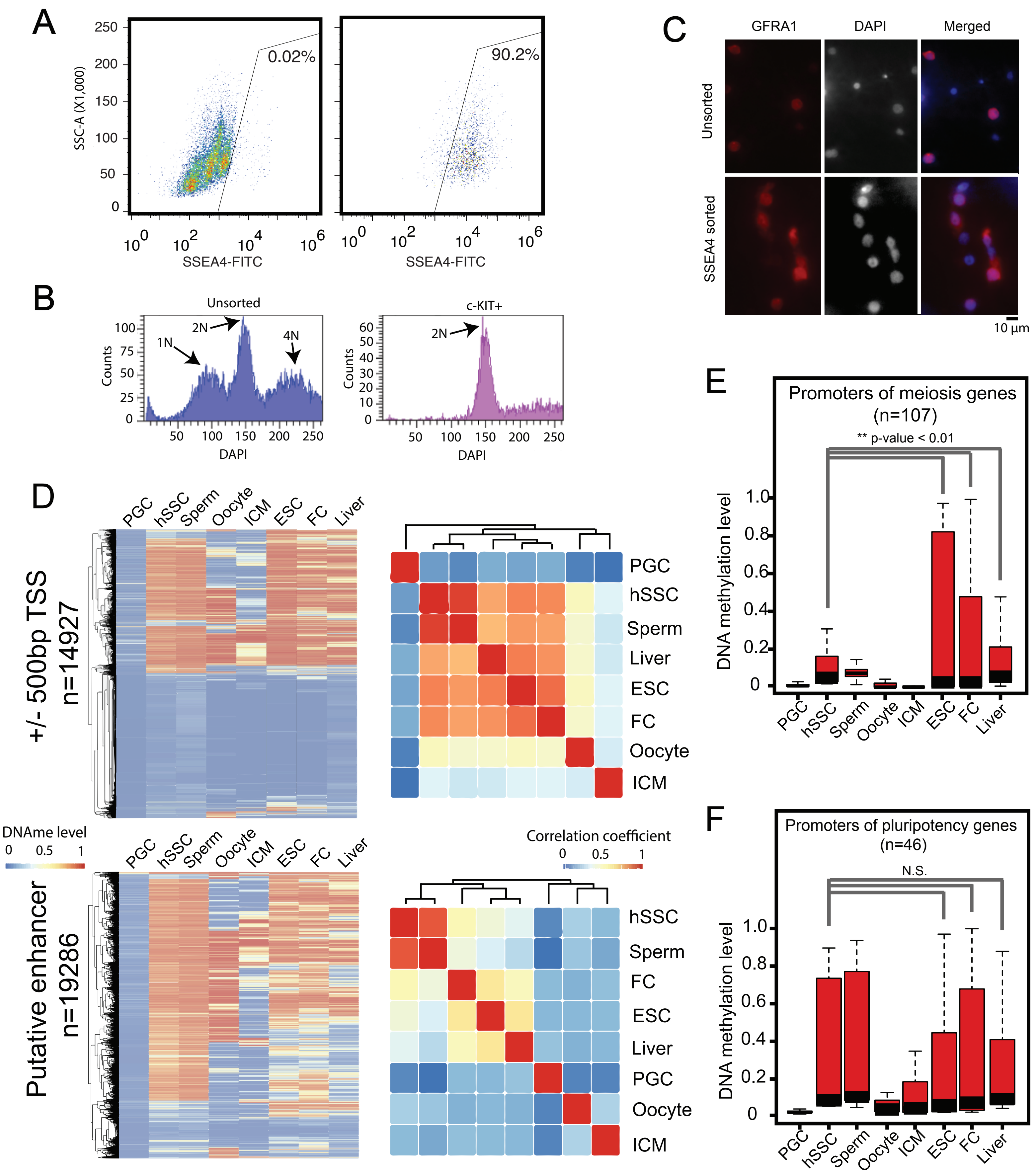

A
SSC-A (X1,000)
0.02%
90.2%
SSEA4-FITC
SSEA4-FITC
B
Unsorted
c-KIT+
1N
2N
4N
Counts
DAPI
C
GFRA1
DAPI
Merged
Unsorted
SSEA4 sorted
10 µm
D
PGC
hSSC
Sperm
Oocyte
ICM
ESC
FC
Liver
+/- 500bp TSS
n=14927
Putative enhancer
n=19286
DNAme level
0 0.5 1
Correlation coefficient
0 0.5 1
E
Promoters of meiosis genes
(n=107)
** p-value < 0.01
DNA methylation level
F
Promoters of pluripotency genes
(n=46)
N.S.
DNA methylation level

Figure S1

Genomic Profiling of hSSCs. Related to Figure 1

(A) Flow cytometry analysis confirming that the SSEA4 MACS sorting procedure enriches for SSEA4+ cells. After MACS sorting, 90.2% cells were SSEA4+. Left: Unsorted control. Right: SSEA4-enriched cells stained by Labeling Checking Reagent-FITC; cells bound by SSEA4-microbeads are stained as FITC+. Y-axis represents Side Scattered Light signal intensity.

(B) Flow cytometry analysis of ploidy number (using DAPI) of unsorted (left) and c-KIT enriched (right) cells. After MACS sorting with the c-KIT antibody, most c-KIT+ cells are diploid (2N), indicating minimal contamination of spermatids (1N) and secondary spermatocytes (4N).

(C) Immunostaining for GFRA1 (hSSC marker) in unsorted (top panels) and SSEA4+ sorted (bottom panels) cells. Following MACS sorting with the SSEA4 antibody, the proportion of GFRA1+ cells increased from 43% to 80%.

(D) Left: hierarchical clustering of average DNA methylation (DNAme) at promoter regions (top) or putative enhancers (bottom) in different tissue types (as indicated on the figure). Right: hierarchical clustering of correlation of average DNAme at promoter regions (top) or putative enhancers (bottom) in different tissue types. Note: promoter regions are defined as regions within +/- 500bp of transcription start sites (TSSs); putative enhancers are defined as regions marked by both H3K4me1 and H3K27ac in human embryonic stem cells (ESCs). Human ESCs H3K4me1 and H3K27ac ChIP-seq data were downloaded from the ENCODE project website (https://www.encodeproject.org). Human DNAme datasets of different human tissues were downloaded from published datasets: human primordial germ cells (PGC) and Liver methylation data from (Guo et al., 2015); Inner Cell Mass (ICM) and Frontal Cortex (FC) methylation data from (Guo et al., 2014); Oocyte methylation data from (Okae et al., 2014); ESC methylation data from (Gifford et al., 2013); human sperm methylation data from (Hammoud et al., 2009).

(E) Boxplots showing average DNAme levels at promoters of 107 meiosis-related genes in different tissues. Gene list from (Guo et al., 2015).

(F) Boxplots showing average DNAme levels at promoters of 46 pluripotency-related genes in different tissues. Gene list from (Guo et al., 2015). N.S.: not significant.

Figure S2

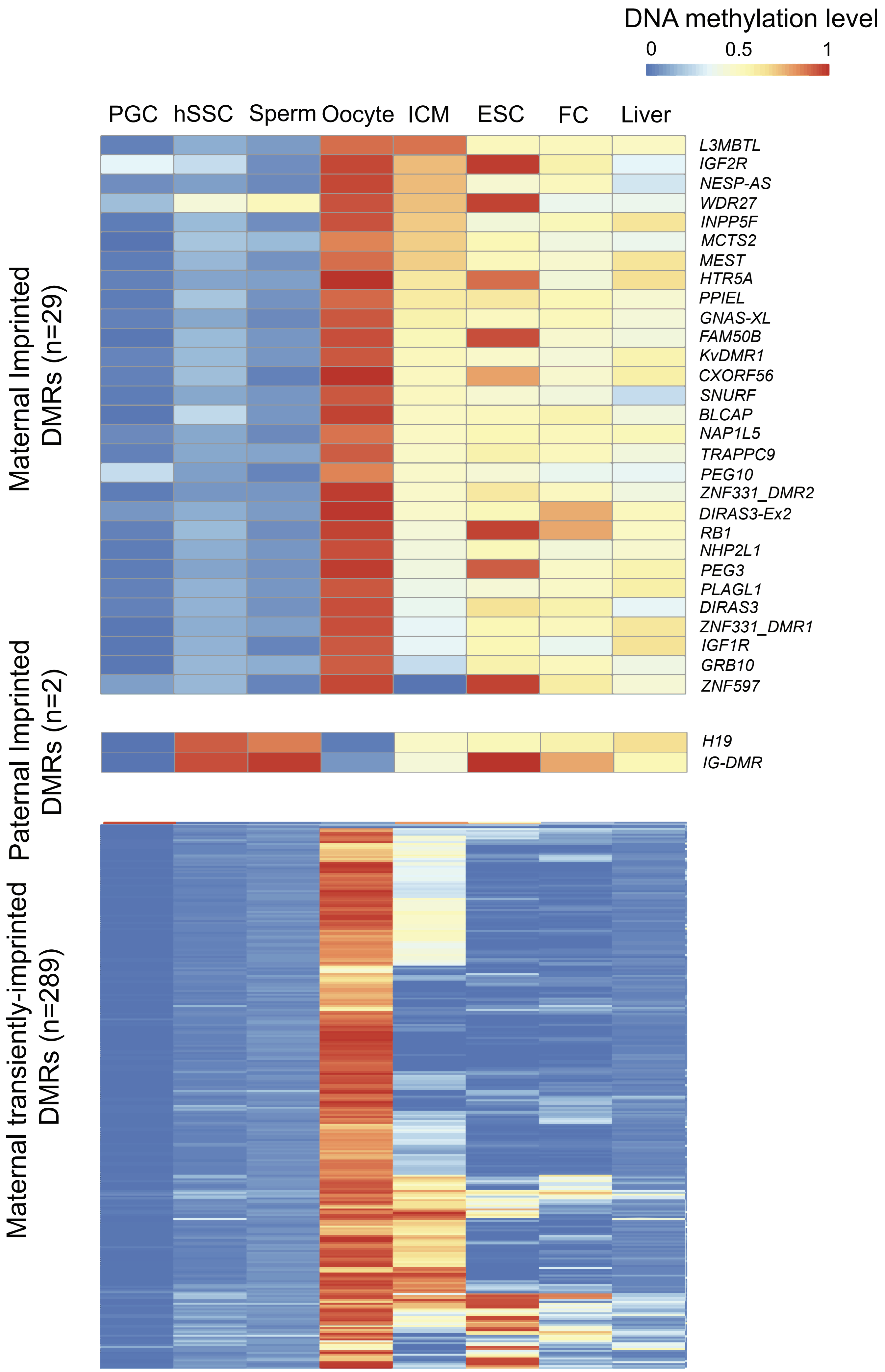
DNA methylation level
0
0.5
1
PGC
hSSC
Sperm
Oocyte
ICM
ESC
FC
Liver
Maternal Imprinted DMRs (n=29)
L3MBTL
IGF2R
NESP-AS
WDR27
INPP5F
MCTS2
MEST
HTR5A
PPIEL
GNAS-XL
FAM50B
KvDMR1
CXORF56
SNURF
BLCAP
NAP1L5
TRAPPC9
PEG10
ZNF331_DMR2
DIRAS3-Ex2
RB1
NHP2L1
PEG3
PLAGL1
DIRAS3
ZNF331_DMR1
IGF1R
GRB10
ZNF597
Paternal Imprinted DMRs (n=2)
H19
IG-DMR
Maternal transiently-imprinted DMRs (n=289)

Figure S2

DNAme Dynamics in Different Tissues at Known Imprinted Sites. Related to Figure 1.

Heatmap showing relative DNAme levels of known imprinted differentially methylated regions (DMRs) in different tissue types. Top: maternal imprinted DMRs (n = 29); middle: paternal imprinted DMRs (n = 2); bottom: maternal transiently-imprinted DMRs (n = 289). Maternal and paternal DMR genomic coordinates are defined in (Okae et al., 2014), and maternal transiently-imprinted DMR coordinates are given in (Pastor et al., 2016).

Figure S3

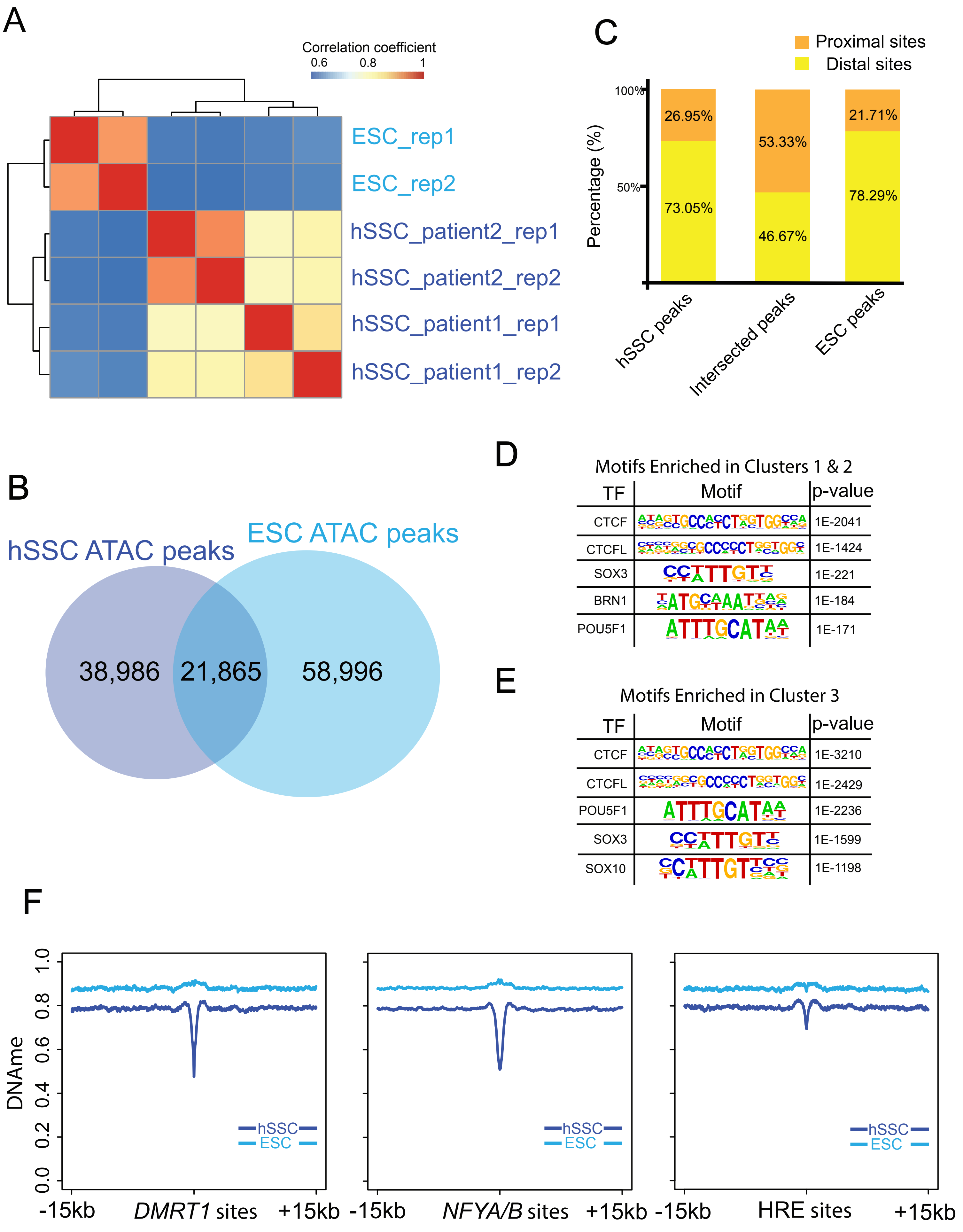

A
Correlation coefficient
0.6
0.8
1
ESC_rep1
ESC_rep2
hSSC_patient2_rep1
hSSC_patient2_rep2
hSSC_patient1_rep1
hSSC_patient1_rep2
C
Proximal sites
Distal sites
100%
50%
Percentage (%)
26.95%
73.05%
53.33%
46.67%
21.71%
78.29%
hSSC peaks
Intersected peaks
ESC peaks
B
hSSC ATAC peaks
ESC ATAC peaks
38,986
21,865
58,996
D
Motifs Enriched in Clusters 1 & 2
TF
Motif
p-value
CTCF 1E-2041
CTCFL 1E-1424
SOX3 1E-221
BRN1 1E-184
POU5F1 1E-171
E
Motifs Enriched in Cluster 3
TF
Motif
p-value
CTCF 1E-3210
CTCFL 1E-2429
POU5F1 1E-2236
SOX3 1E-1599
SOX10 1E-1198
F
DNAme
0.0
0.2
0.4
0.6
0.8
1.0
hSSC
ESC
-15kb
DMRT1 sites
+15kb
-15kb
NFYA/B sites
+15kb
-15kb
HRE sites
+15kb

Figure S3

Comparison of ATAC-seq Signals Between hSSCs and ESCs. Related to Figure 2.

(A) Hierarchical clustering of correlation between two different technical replicates for ESC and hSSC (from 2 different patients) as indicated on the figure. Note the color scale bar represents correlation between 0.6-1.

(B) Venn diagram of ATAC-seq peaks shared between hSSCs and ESCs. See Methods for peak calling thresholds.

(C) Relative distribution of proximal or distal sites in SSEA4+ hSSC peaks, ESC peaks and the shared/intersected peaks that were defined in Figure S3B.

(D) Motifs enriched in the peaks from Clusters 1 & 2 (from main Figure 2A). Motifs were found using findMotifGenome.pl application (v4.8.3, homer).

(E) Motifs enriched in the peaks from Cluster 3 (from main Figure 2A). Motifs were found using findMotifGenome.pl application (v4.8.3, homer).

(F) Metaplots of relative DNA methylation levels within 15kb around *DMRT1*, *NFYA/B* and HRE binding sites, in hSSCs (dark blue) and ESCs (light blue).

Figure S4

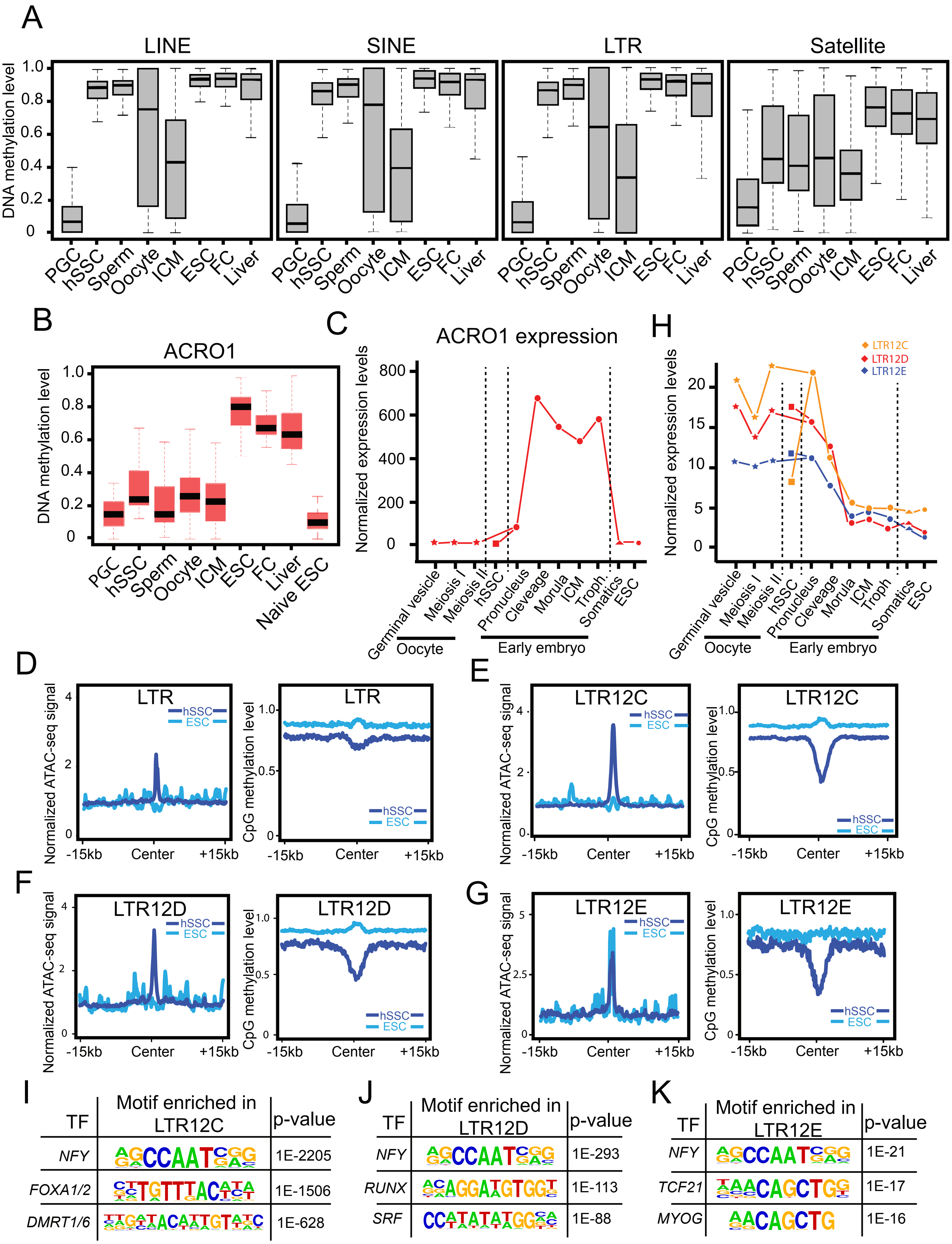
A
LINE
SINE
LTR
Satellite
DNA methylation level
1.0
0.8
0.6
0.4
0.2
0
PGC
hSSC
Sperm
Oocyte
ICM
ESC
FC
Liver
B
ACRO1
DNA methylation level
Naive ESC
C
ACRO1 expression
Normalized expression levels
800
600
400
200
Germinal vesicle
Meiosis I
Meiosis II
hSSC
Pronucleus
Cleveage
Morula
ICM
Troph.
Somatics
ESC
Oocyte
Early embryo
H
LTR12C
LTR12D
LTR12E
Normalized expression levels
20
15
10
5
Troph
D
LTR
hSSC
ESC
Normalized ATAC-seq signal
CpG methylation level
-15kb
Center
+15kb
E
LTR12C
F
LTR12D
G
LTR12E
I
TF
Motif enriched in LTR12C
p-value
NFY
1E-2205
FOXA1/2
1E-1506
DMRT1/6
1E-628
J
Motif enriched in LTR12D
NFY
1E-293
RUNX
1E-113
SRF
1E-88
K
Motif enriched in LTR12E
NFY
1E-21
TCF21
1E-17
MYOG
1E-16

Figure S4

Expression, DNAme and ATAC-seq Signals at Repetitive Elements. Related to Figure 2.

(A) Boxplots of relative DNAme levels in different tissues at several repetitive elements (LINE, SINE, LTR and Satellite), as indicated on top of each panel.

(B) Boxplots of relative DNAme levels at ACRO1 elements in different tissues.

(C) Expression levels of ACRO1 elements at different stages of development of human oocytes, hSSCs, early embryos, somatic cells and ESCs.

(D-G) Metaplots of ATAC-seq signals (left) and relative levels of DNA methylation (right) in hSSCs (dark blue) and ESCs (light blue) in the 15kb regions flanking LTR12 elements (D), LTR12C elements (E), LTR12D elements (F) and LTR12E elements (G).

(H) Expression levels of LTR12C, LTR12D and LTR12E at different developmental stages in oocytes, hSSCs, early human embryos, somatic cells and ESCs.

(I) Motifs enriched in LTR12C sites, using findMotifGenome.pl application (v4.8.3, homer)

(J) Motifs enriched in LTR12D sites, using findMotifGenome.pl application (v4.8.3, homer)

(K) Motifs enriched in LTR12E sites, using findMotifGenome.pl application (v4.8.3, homer)

Figure S5

A

	SSEA4+ #1	SSEA4-/c-KIT+ #1	SSEA4+ #2	SSEA4-/c-KIT+ #2	SSEA4+ #3	SSEA4-/c-KIT+ #3	c-KIT+ #3	c-KIT-/SSEA4+ #3
Pearson correlation	0.993	0.988	0.991	0.979	0.968	0.951	0.972	0.964

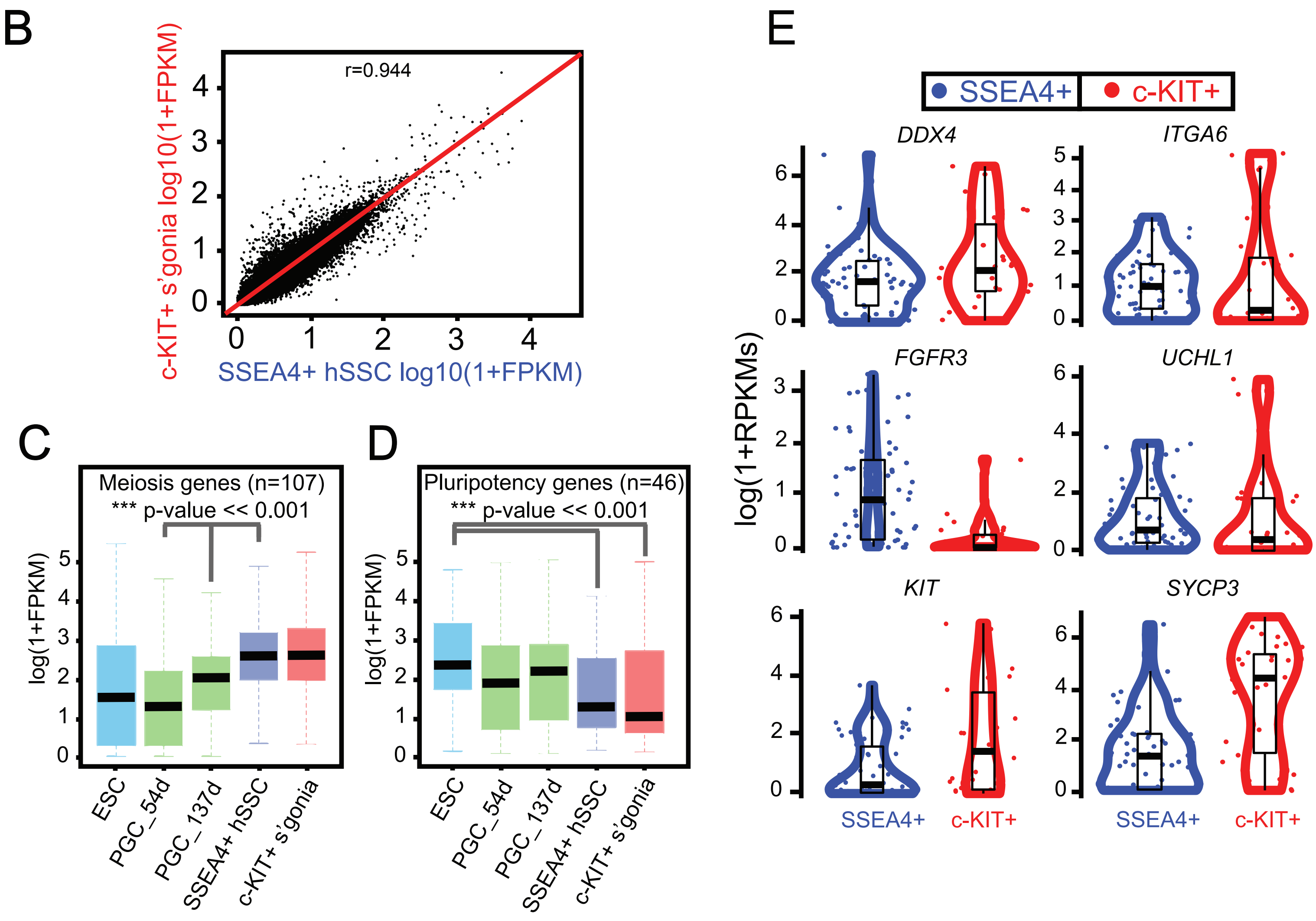

Figure S5

Profiling of Transcriptome (RNA-seq) in hSSCs. Related to Figure 3 and Figure 4.

(A) Pearson correlation of the bulk transcriptome data (RNA-seq) between the two technical replicates for the 3 testicular samples from each MACS selection (SSEA4+ or c-KIT+); the observed correlations demonstrate low technical variation. Note: given the high correlation, technical replicates were combined for subsequent computational analysis.

(B) Scatterplot comparing gene expression levels (FPKM) in SSEA4+ hSSCs versus c-KIT+ spermatogonia, showing high correlation between expression levels (r = 0.944).

(C) Boxplots describing the expression levels of 107 meiosis related genes in ESCs, early PGCs (54 days), later PGCs (137 days), SSEA4+ hSSCs and c-KIT+ spermatogonia. Gene list from (Guo et al., 2015).

(D) Boxplots describing the expression levels of 46 pluripotency related genes in ESCs, early PGCs, later PGCs, SSEA4+ hSSCs and c-KIT+ spermatogonia. Gene list from (Guo et al., 2015).

(E) Violin- and box-plots of expression levels for selected key marker genes in SSEA4+ (blue) or c-KIT+ (red) single cells. Each dot represents the expression level within a single cell for the gene indicated on top of each panel.

Figure S6

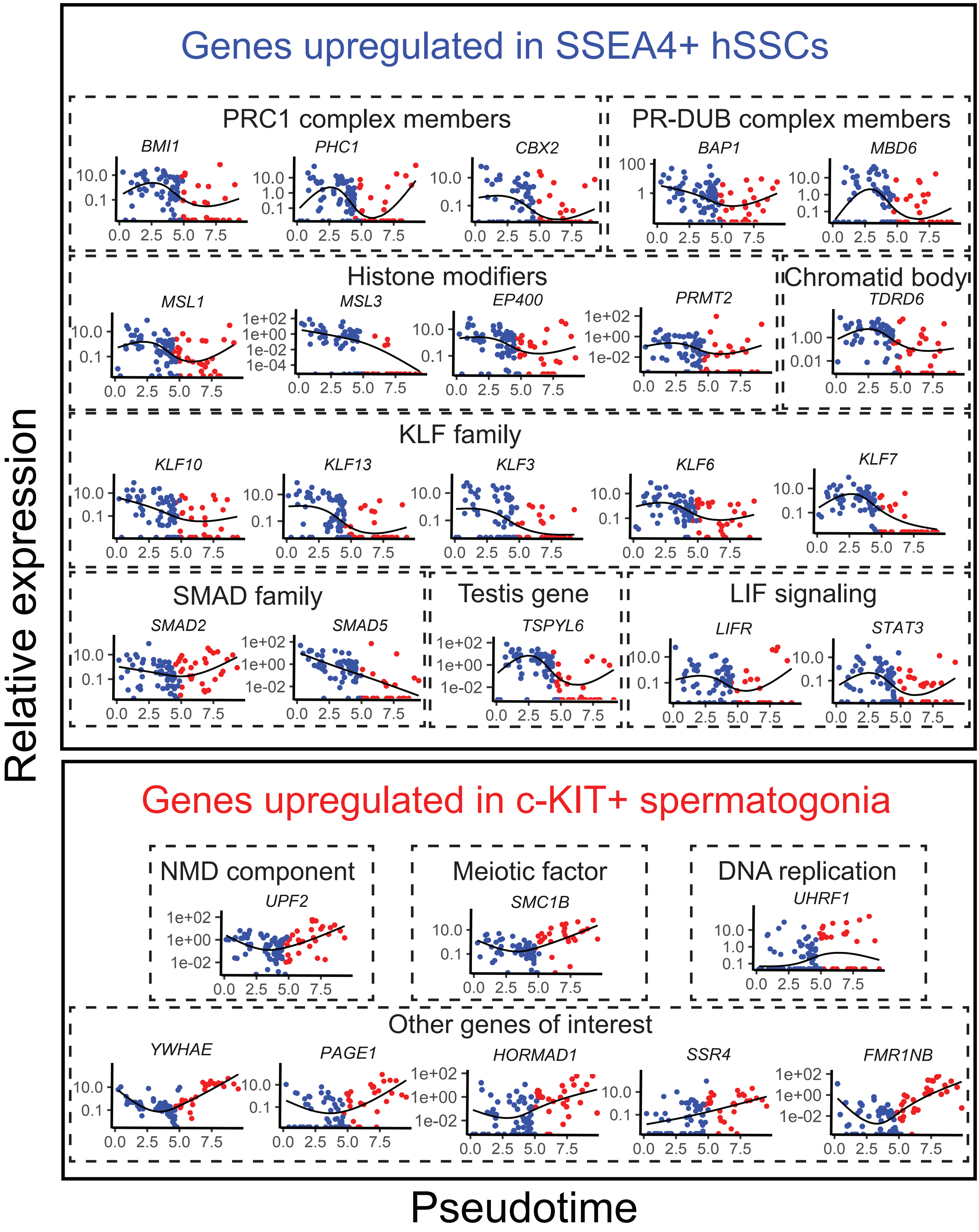
Genes upregulated in SSEA4+ hSSCs
PRC1 complex members
BMI1
PHC1
CBX2
PR-DUB complex members
BAP1
MBD6
Histone modifiers
MSL1
MSL3
EP400
PRMT2
Chromatid body
TDRD6
KLF family
KLF10
KLF13
KLF3
KLF6
KLF7
SMAD family
SMAD2
SMAD5
Testis gene
TSPYL6
LIF signaling
LIFR
STAT3
Genes upregulated in c-KIT+ spermatogonia
NMD component
UPF2
Meiotic factor
SMC1B
DNA replication
UHRF1
Other genes of interest
YWHAE
PAGE1
HORMAD1
SSR4
FMR1NB
Relative expression
Pseudotime

Figure S6

Expression of Representative Selected Key Genes along ‘Pseudotime’ in SSEA4+ and c-KIT+ single cells. Related to Figures 4-6.

Depicted selected genes are grouped into categories/pathways of interest and individual panels represent the relative expression levels of single SSEA4+ (blue) or c-KIT+ (red) cells projected along ‘pseudotime’ (on x-axis). Note: the data is depicted as compressed (log10) transformed expression data (on y-axis), and as expected of typical single-cell datasets, ~30-70% of single cells provide non-zero expression of individual genes. See also main text, and legends of Figures 4-6 for further descriptions of pseudotime plots.

Figure S7

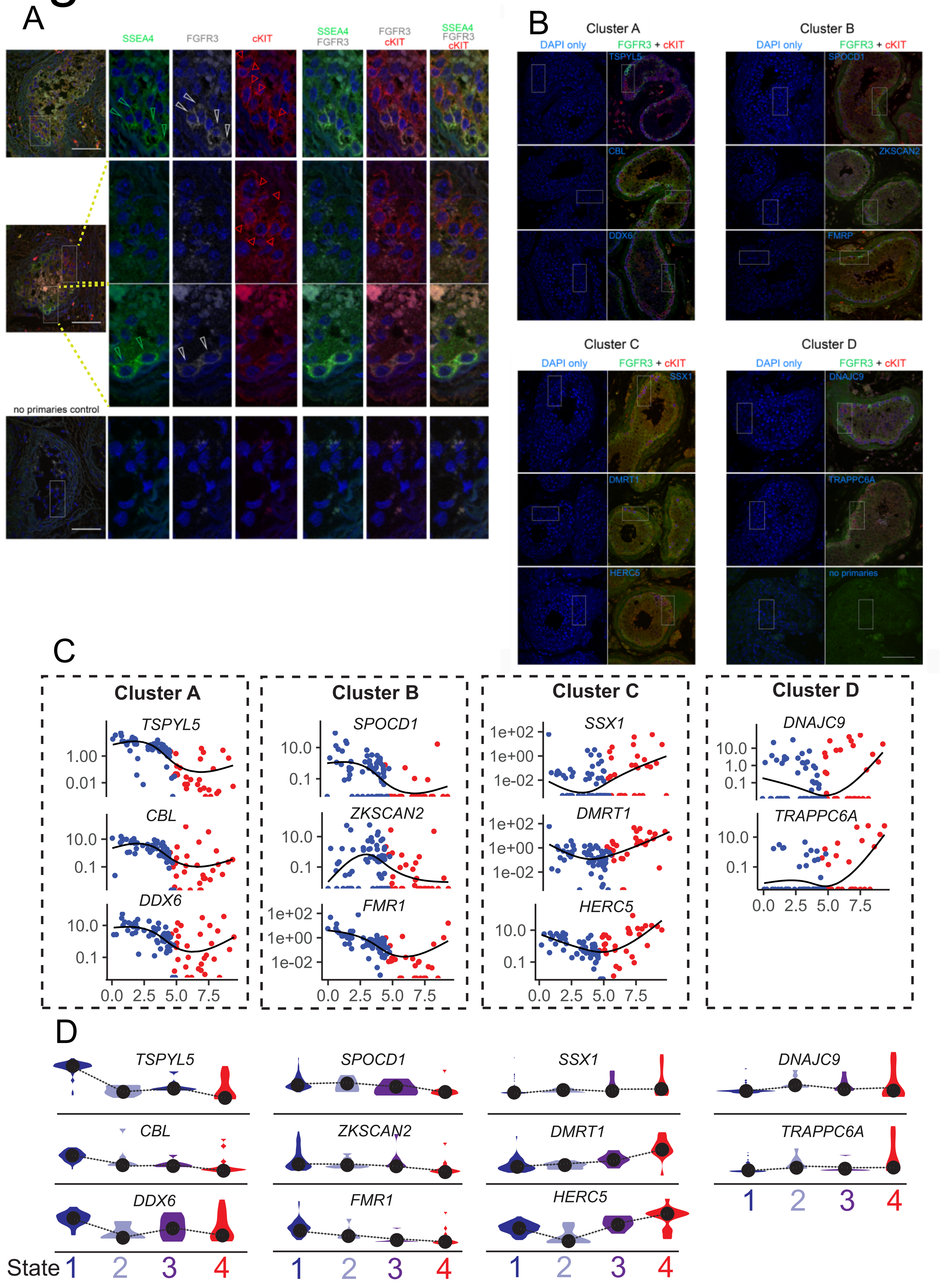
A
SSEA4
FGFR3
cKIT
SSEA4 FGFR3
FGFR3 cKIT
SSEA4 FGFR3 cKIT
no primaries control
B
Cluster A
DAPI only
FGFR3 + cKIT
TSPYL5
CBL
DDX6
Cluster B
SPOCD1
ZKSCAN2
FMRP
Cluster C
SSX1
DMRT1
HERC5
Cluster D
DNAJC9
TRAPPC6A
no primaries
C
Cluster A
TSPYL5
CBL
DDX6
Cluster B
SPOCD1
ZKSCAN2
FMR1
Cluster C
SSX1
DMRT1
HERC5
Cluster D
DNAJC9
TRAPPC6A
0.0 2.5 5.0 7.5
D
TSPYL5
CBL
DDX6
SPOCD1
ZKSCAN2
FMR1
SSX1
DMRT1
HERC5
DNAJC9
TRAPPC6A
State 1 2 3 4

Figure S7

Direct Visualisation of Protein Expression by Immunofluorescence Validate Predictions from the Genomics Data Clustering. Related to Figure 7

(A) Immunolocalization of the SSEA4 glycolipid, FGFR3 and c-KIT proteins on frozen sections of human seminiferous tubules.

The protein expression of FGFR3 (white), a Cluster A marker, overlaps with that of SSEA4 (green), while c-KIT (red) is expressed in cells that are SSEA4- and FGFR3-. The areas boxed in the 2 seminiferous tubules presented on the left side of the figure with 4 color channels) are the regions that are seen at high magnification in columns 2 to 7. Above the panels, a colored annotation has been added to account for the antigens that are visualised in single- (columns 2-4), double- (columns 5-6) or triple- (right column 7) color channels, in combination with nuclear staining revealed by DAPI (blue). Green arrowheads point to cells expressing SSEA4; white arrowheads point to the same cells when they are visualised for FGFR3 expression; small red arrowheads point to c-KIT expressing cells – these cells do not express SSEA4 or FGFR3.

The bottom row represents the negative controls – no primary antibodies were added and they are visualised in the same channels as the annotation on top of the columns. The white bar is 100um.

(B) Low magnification of the FFPE tubular cross-sections represented in Figure 7. In each case, the left panels represent the nuclear staining only of the tubule, seen using DAPI (blue) and the right panel shows the triple antibody co-immunostaining. Triple immunolocalization was performed using FGFR3 (Cluster A marker used as a surrogate for SSEA4, in green), c-KIT (Cluster D marker, in red) and one of the 11 different cluster-specific antigens (in blue) on FFPE sections of human seminiferous tubules. For each cluster, two (Cluster D) or three (Clusters A-C) antigens were selected on the basis of the quality of the published staining in the Human Protein Atlas. Cluster-specific antigen's names are indicated in blue on the corresponding panel. The boxed region represents the part of the tubule shown at higher magnification on Fig 7. The bottom right panels represent the negative (no primary) controls. All pictures are at the same magnification and the white bar in the bottom right panel is 100um.

(C) Pseudotime plots of the 11 genes encoding the antigens for which the immunostainings are shown in Figure 7 and Figure S7B. The immunostaining results obtained for the 11 antigens are consistent with the prediction of the single cell data: as shown on panels A and B (for Clusters A-B), the pseudotime profile resembles that of *FGFR3* (see Figure 4D, left) , while in Panels C and D (for Clusters C-D) the trend line mirrors that seen on the *KIT* pseudotime plot (see Figure 4D, right). Pseudotime plots for *FGFR3* and *KIT* are presented in Figure 4D. Note: FMRP is the protein product of *FMR1*.

(D) Violin plots of the 11 genes encoding the antigens for which the immunostainings in Figure 7 and Figure S7B were performed, representing each gene relative expression levels in the four cellular States (as decribed in the main text and on Figure 6C-D). Y-axis represents the Z-score of expression levels. The median levels representative of each State have been linked by a dotted line to depict the developmental trajectory.
